# Quaternary low-temperature serpentinization and carbonation in the New Caledonia ophiolite

**DOI:** 10.1038/s41598-023-46691-y

**Published:** 2023-11-08

**Authors:** Marianna Corre, Fabrice Brunet, Stéphane Schwartz, Cécile Gautheron, Arnaud Agranier, Stéphane Lesimple

**Affiliations:** 1 ISTerre, Université Grenoble Alpes, USMB, CNRS, IRD, 38041 Grenoble, France; 2 GEOPS, Université Paris-Saclay, CNRS, 91405 Orsay, France; 3 GEO-OCEAN, Université de Bretagne Occidentale, IUEM, CNRS, 29280 Plouzané, France; 4 Service Géologique de Nouvelle-Calédonie, BP M2, 98849 Nouméa, New Caledonia

**Keywords:** Solid Earth sciences, Geochemistry, Mineralogy, Petrology, Carbon capture and storage, Hydrogen energy

## Abstract

The low-temperature alteration (< 150 °C) of ophiolites by infiltrated meteoric waters removes atmospheric CO_2_ through mineral carbonation and is assumed to generate H_2_ and possibly CH_4_ according to so-called serpentinization reactions. This overall alteration pattern is primarily constrained by the chemical composition of alkaline springs that are issued in several ophiolites worldwide. Here we report on the fingerprint, as veinlet mineralization, of the reactive percolation of such meteoric waters in the New Caledonia ophiolite (Massif du Sud). The mineralization which resulted from carbonation and serpentinization reactions, is young (< 2 Ma) and formed at a temperature of ca. 95 °C. It is mainly composed of lizardite, dolomite, magnetite ± pyroaurite. Thermochemical simulation of mineral–water equilibria shows that the percolating aqueous fluid was alkaline and H_2_ bearing. The δ^13^C of dolomite is exceptionally high, between 7.1 and up to 17.3‰, and is interpreted as evidence of low-temperature methanogenesis. Overall, the percolating fluid had a chemical composition similar to that of the waters issued today in the (hyper)alkaline springs of the Massif du Sud. The studied veinlets are thus interpreted as a sample of the plumbing system that fed an ancient Quaternary alkaline spring in the area.

## Introduction

The interaction between ultramafic rocks and natural aqueous fluids triggers two important classes of reactions. (1) Serpentinization reactions produce hydrated phases such as serpentine group minerals, ideally ((Mg,Fe)_3_Si_2_O_5_(OH)_4_), and brucite ((Mg,Fe)(OH)_2_), as well as magnetite (Fe_3_O_4_) which is involved in the development of the mesh texture. (2) Carbonation reactions lead to the crystallization of Mg–Ca carbonates, mainly dolomite and magnesite^1^.

Serpentinization reactions are particularly efficient on Earth at slow and ultra-slow mid-oceanic ridges where large portions of the oceanic mantle are exposed to hydrothermal fluids^2,3^. In addition to serpentine minerals, the aqueous fluids/peridotite interaction produces alkaline fluids (i.e. of high pH) containing H_2_ and CH_4_ gases^4–7^ which represent a possible energy source for sustaining microbial activity in submarine hydrothermal vents^8,9^ and, possibly, for some of the earliest life^10^. Carbonate veins hosted in ultramafic rocks drilled at the Mid Atlantic Ridge show that carbonation reactions also occur as the result of fluid-rock interaction^11^.

Calcium-rich alkaline and hyper-alkaline (pH > 11) springs bubbling H_2_ and CH_4_ are also encountered on land in several ophiolites worldwide (Philippines, Oman, New Zealand, New Caledonia, and Turkey)^12^ where partially serpentinized ultramafic rocks interact chemically with meteoric waters^13^. Based on their chemical and isotopic composition, these waters have been interpreted as resulting from present-day serpentinization^14^ at low temperatures (LT), typically in the 85–115 °C range^15^. The commonly admitted model of ophiolite LT alteration^16–20^ by meteoric water involves both serpentinization and carbonation. In a first alteration step at shallow levels, groundwater acquires Mg^2+^ and HCO_3_^−^ rich compositions (Type I waters)^20^. In a further step of interaction under sub-surface conditions, alkaline Ca-OH rich waters (Type II)^20^ with relatively high *f*H_2_ are produced due to the precipitation of Mg-carbonates and LT serpentinization products. These alkaline waters (Type II) when they reach the surface, precipitate Ca-carbonates by reaction with atmospheric CO_2_^21,22^. Overall, the alteration of peridotite massifs is a natural way to remove CO_2_ from the atmosphere. In the Samail ophiolite (Oman), the rate of atmospheric CO_2_ conversion into solid carbonates is estimated at 10^4^^–^10^5^ tons per year^1^. While ophiolites can be considered greenhouse gas (GHG) sinks through mineral carbonation, H_2_ produced by LT serpentinization might promote the formation of CH_4_, another important GHG, through abiotic CO_2_ reduction or chemoautotrophic biochemical processes. However, it has been shown that the amount of CH_4_ outgassed at the Samail ophiolite (Oman) is unlikely to offset negative carbon emissions estimated from active carbon mineralization reactions^23^.

Despite the relevance of LT serpentinization and associated carbonation in terms of planet habitability, gas production and emission (H_2_, CH_4_), and CO_2_ mineral sequestration, the mineralogical processes at play under subsurface temperature conditions remain poorly understood^24^. RedOx reactions involving Fe-bearing minerals and water, schematically 2Fe(II)O + H_2_O = Fe(III)_2_O_3_ + H_2_, are the best candidates for abiotic H_2_ production^25^. However, the Fe-bearing minerals involved in low-temperature serpentinization (T < 150 °C) and their phase relationships still need to be determined, e.g., residual olivine, Fe-brucite or serpentine as Fe(II) source and magnetite, Fe(III)-bearing serpentine or hydroxides as oxidation products. Indeed, it should be recalled that the collection of meaningful experimental data on LT serpentinization and associated H_2_ and CH_4_ production is highly difficult^26^.

**Figure 1:**
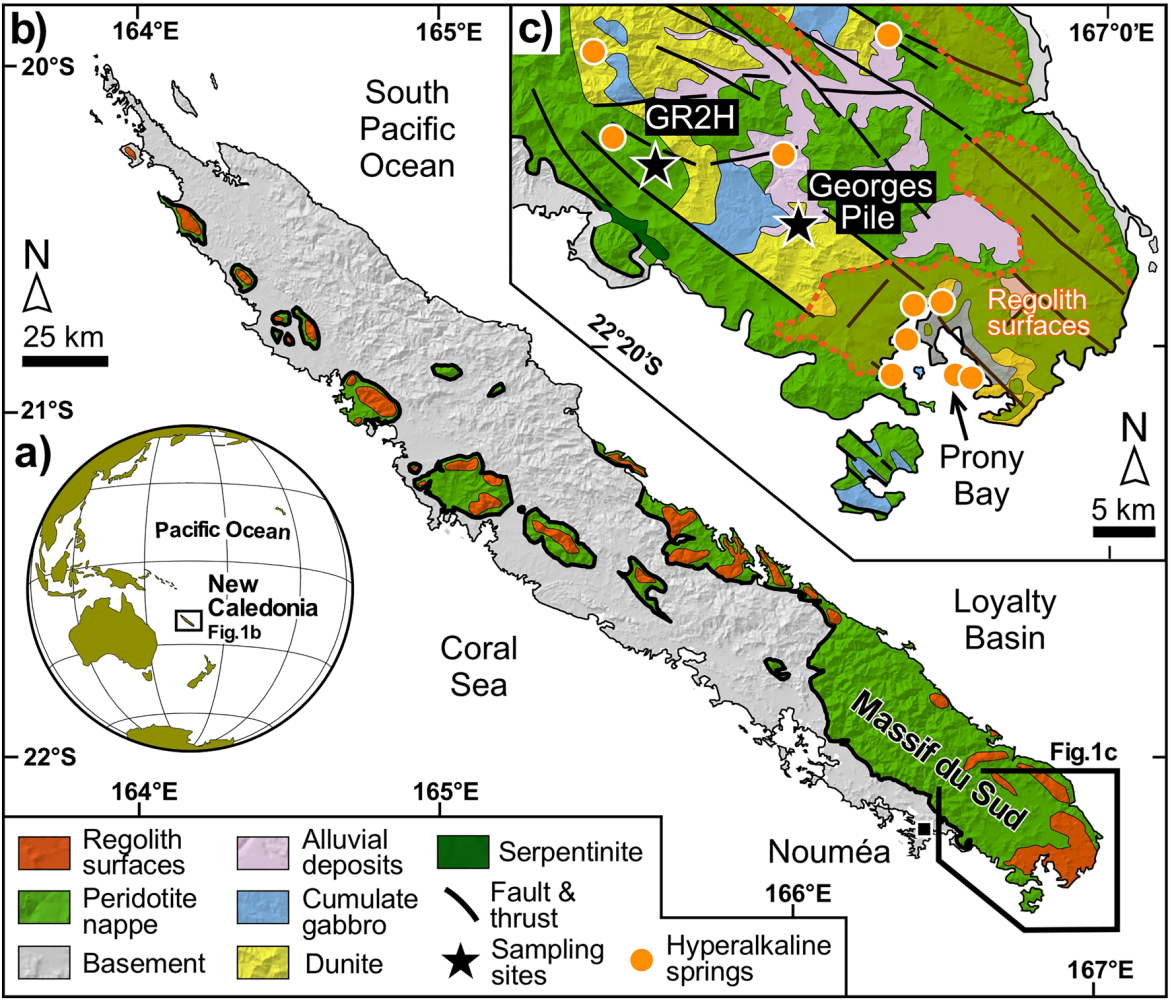
Sample location. (**a**) New Caledonia position in the Pacific Ocean. (**b**) Simplified geological map of Grande Terre, featuring only the peridotite nappe and the regolith. (**c**) Simplified geological map of the southern part of the Massif du Sud with location of the studied samples (GR2H and Georges Pile) and hyper-alkaline springs (modified from Maurizot et al.^30^).

We show here that such LT phase relationships are preserved in veinlet mineralization from the New Caledonia ophiolite (Massif du Sud) occurring near hyperalkaline springs (Fig. 1) where H_2_ and CH_4_ are currently venting. These millimeter-sized veinlets crosscut and thus postdate an early mesh texture preserved in the partially serpentinized host-peridotite. They contain typical serpentinization products, lizardite, a serpentine-group mineral, magnetite, along with carbonates, abundant dolomite (CaMg(CO_3_)_2_), and pyroaurite (Mg_6_Fe_2_(OH)_16_(CO_3_),4.5H_2_O).

In the present study, mineral composition and textures were characterized, and the composition of the water from which these minerals precipitated was inferred from thermochemical modeling. In situ δ^18^O and δ^13^C data were collected on dolomite–magnetite pairs for thermometry. Submillimeter-sized magnetite crystals found in these veins were dated using magnetite (U-Th)/He geochronology (MgHe)^27,28^ to constrain the fluid percolation timing.

## Geological setting and sample description

The New Caledonia ophiolite is located in the South-West Pacific Ocean at the Grand Terre, the main island of New Caledonia (500 km length), formed by obduction of the Loyalty oceanic basin onto the continental crust of the Norfolk Ridge (Fig. 1a) during the late Eocene (ca. 34 Ma)^29^. The ophiolite covers one-third of the island (Fig. 1b) and corresponds to a nappe of peridotite 1.5–3.5 km thick^30^. The sole of the peridotite nappe is 20–400 m thick and is composed of strongly deformed serpentinite. It is expected to be a zone of preferential fluid circulation^31^. The central body of the peridotite nappe consists of partially serpentinized peridotites (from 20 to 80 vol.%) developed by a dense fracture system with mesh-type serpentinization characteristic of oceanic alteration^32^. The uppermost part of the peridotite nappe is deeply weathered and forms a regolith (Fig. 1c) of variable thickness (< 100 m) whose development began at the late Oligocene, at least 25 Ma ago^33^. In the northern part of New Caledonia (Koniambo massif), Mg-carbonate veins are related to the circulation of meteoric fluids from the surface to the peridotite sole. The corresponding drainage system is expected to be contemporaneous with tectonic events (obduction and post-obduction) that occurred before 20 Ma and may have produced lateritization^31^.

The Georges Pile (− 22.115° N/166.431° E) and GR2H (− 22.206° N/166.628° E) localities were selected in this study for the occurrence of euhedral magnetite with sizes above 400 µm, suitable for dating (see “Methods”). The two localities are separated by 15 km from each other (Fig. 1c; Figs. S1 and S2) and correspond to former chromium mines. They belong to the main ultramafic unit of the Massif du Sud, which is composed mainly of harzburgite (> 85%) associated with dunite and cumulate gabbros^34,35^. This area is known for its H_2_- and CH_4_-rich hyperalkaline springs (Fig. 1c) at *La rivière des Kaoris*, *Carénage Bay*, and *Prony Bay*^36–38^. Samples from the GP area (Fig. 1c) were collected in a zone of preferential aqueous fluid circulation (ca. 30 cm thickness) at the interface between a plagiogranite dyke dated from 27 to 24 Ma^39^ and the host serpentinized dunite (Fig. S1). Contrary to GP samples, the rock samples collected at GR2H (Fig. S2) are preserved from weathering and were thus suitable for retrieving the conditions of magnetite formation.

## Results

### Sample texture and mineralogy

#### Georges Pile samples

Samples from the GP area (Fig. 1c) contain flattened magnetite crystals with euhedral morphology and a diameter of up to 600 µm. Minor element distribution (Mn, Ni, Fe, Mg, and Co) in magnetite outlines remarkable growth zones (Fig. S3). The magnetite-bearing zone is mainly composed of lizardite (+/− chrysotile), and olivine is no longer present. The magnetite phase relationships are mostly obliterated by a strong weathering which is characterized by the formation of kaolinite filling in voids. Despite weathering, magnetite (and magmatic Cr-spinel) are preserved, but they are surrounded by an oxidation corona composed of goethite. Beyond the serpentinized magnetite-bearing alteration zone (Fig. S1), the host dunite is also serpentinized with around 20 wt.% residual olivine. Lizardite is the dominant serpentine mineral which co-exists with ferroan brucite or pyroaurite (x_Fe_ ~ 0.2), magnetite and Cr-spinel, ((Mg_0.45_,Fe_0.55_)(Cr_0.62_,Al_0.33_,Fe_0.05_)_2_O_4_).

#### GR2H samples

**Figure 2:**
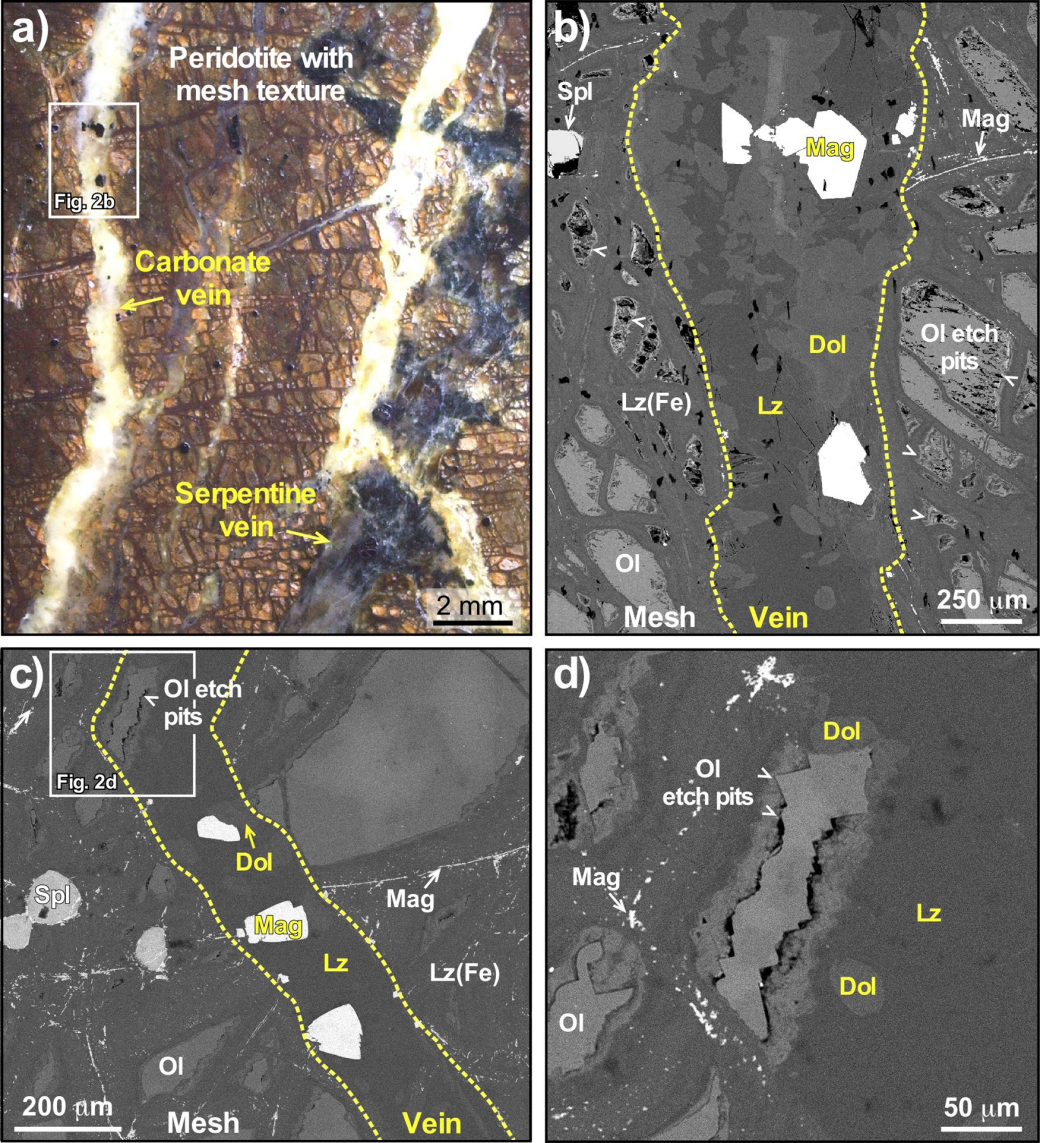
Optical and SEM images of the GR2H sample. (**a**) Optical image (reflected mode) of the carbonate veins mainly composed of dolomite, serpentine and magnetite (GR2H); (**b**) SEM image (BSE mode) showing an example of veinlet mineralization. Euhedral magnetite grains are visible in the core of the vein along with dolomite and Fe-poor lizardite; (**c**) SEM image (BSE mode) of another veinlet including an olivine grain with pronounced dissolution features (etch pits); (**d**) blow-up of the olivine grain from the veinlet displayed in (**c**)—Lz, Fe-poor lizardite; Lz(Fe), Fe-bearing lizardite; Dol, dolomite, and Ol, olivine. Magnetite (Mag) in mesh, spinel grains (Spl) and olivine etch-pits are highlighted by arrowheads.

Samples collected at GR2H (Fig. S2) show a network of late milky veins with widths less than 1 mm (Fig. 2), which are found to either crosscut or follow the mesh texture. The veins are filled with calcian dolomite (Mg/Ca = 0.74) and Fe-poor lizardite (FeO < 1.5 wt.%, Mg# = 0.98, Supplementary Table S1). The thickest veins, up to 2 mm in size, contain subhedral to euhedral magnetite crystals with sizes up to 500 µm. They show, like GP magnetite, remarkable chemical zoning (Fig. S4). With a Ti concentration below 400 ppm and a Ni/Cr ratio above 50 (Supplementary Table S1), magnetite from both occurrences falls at the end of the “*hydrothermal magnetite”* field defined by Dare et al.^40^ and within the compositional range of magnetite formed by serpentinization reactions^40^.

Textural relationships in GR2H samples indicate that calcian dolomite, magnetite, and Fe-poor lizardite co-crystallized. Contacts between magnetite, dolomite and serpentine are sharp (Fig. S5a). Dolomite and serpentine grew on magnetite crystal edges, but they are also found included within magnetite (Fig. S5a). Furthermore, intergrowths of pyroaurite—lizardite + /− dolomite are found in close contact with euhedral magnetite at the vein wall (Fig. S6). Both serpentinization degree (up to 60%) and olivine alteration features (i.e. pronounced etch-pits) gradually increase towards the dolomite-bearing veins (Fig. 2c and d). Carbonate grains (< 50 µm) are found in the zones of intense olivine alteration (Fig. S5b).

### Magnetite (U-Th)/He dating and δ^18^O/δ^13^C isotopic analyses

Magnetite crystals from GP and GR2H were selected for (U-Th)/He geochronology. A total of five MgHe ages on hand-picked magnetite crystals from GP (n = 3) and GR2H (n = 2) were obtained, with MgHe ages ranging from 0.5 ± 0.1 to 0.7 ± 0.2 Ma and 1.2 ± 0.3 to 1.5 ± 0.3 Ma, respectively (Supplementary Table S2, Fig. S8).

**Figure 3:**
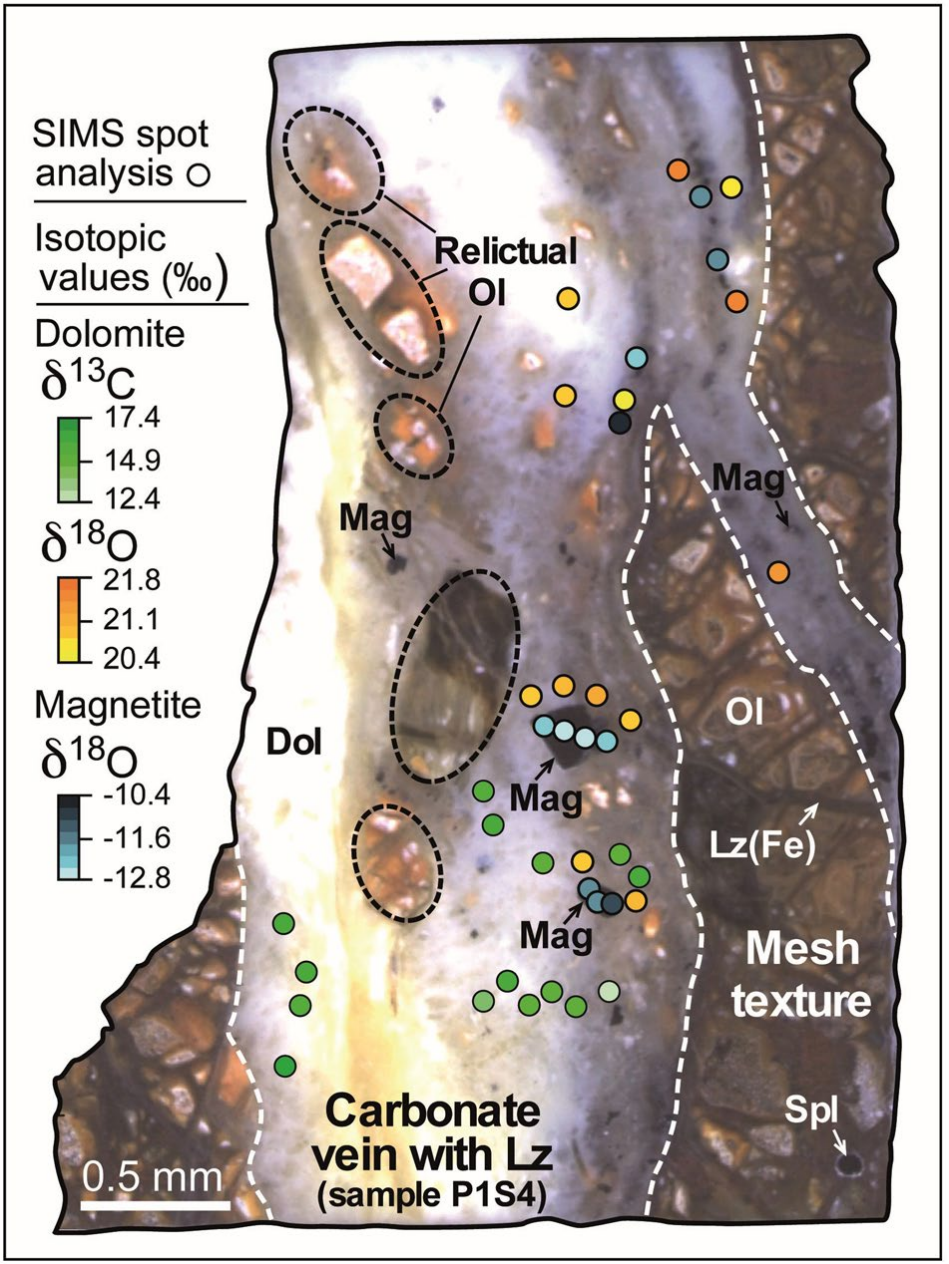
Location of the δ^13^C (dolomite, green circles) and δ^18^O data points (dolomite, orange circles; magnetite, blue circles) in GR2H sample. Symbols are as in Fig. 2. Relictual olivine corresponds to partly altered olivine.

**Figure 4:**
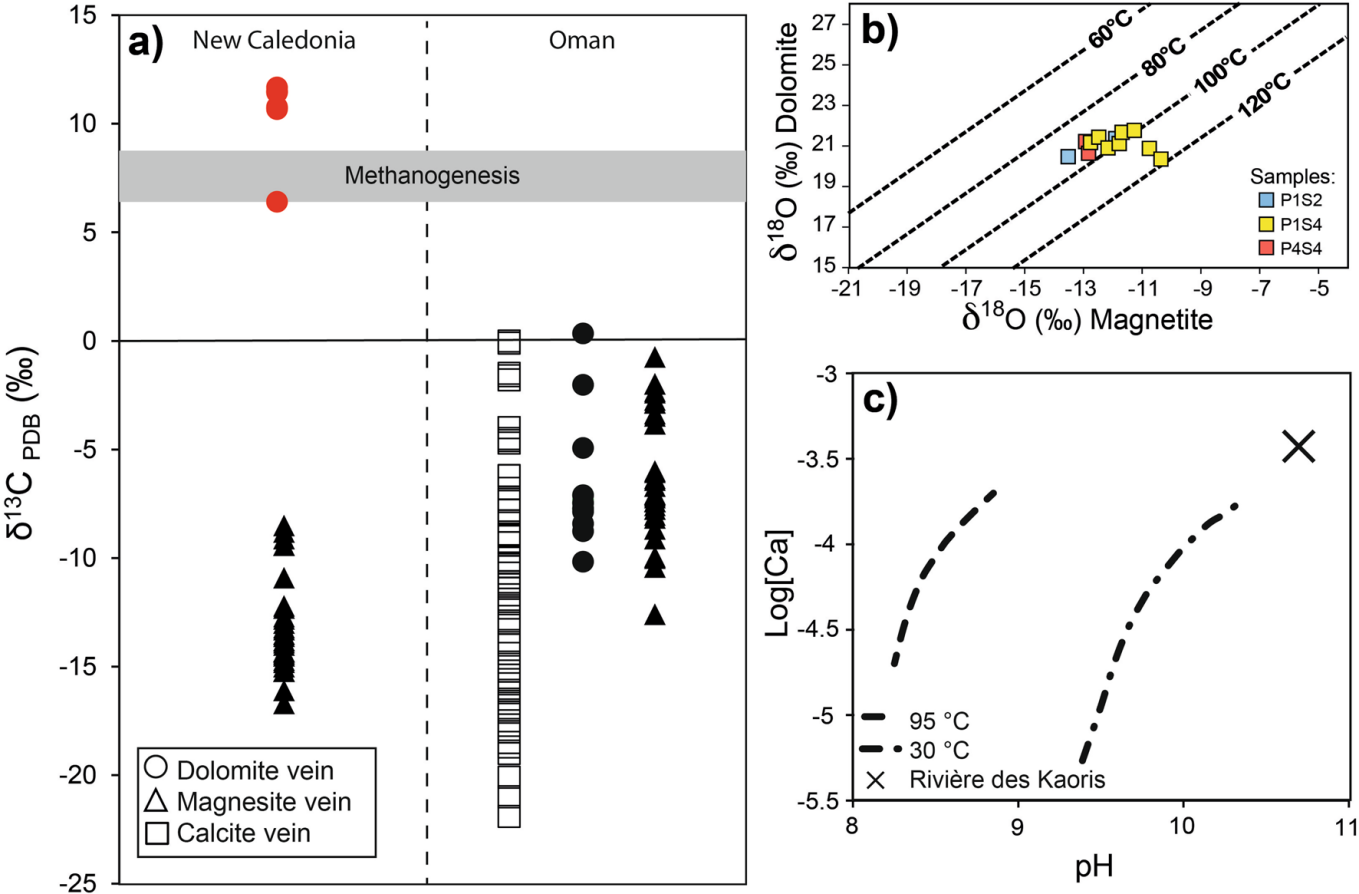
Carbon and oxygen isotopic data, crystallization temperature (GR2H sample), and pH simulation. (**a**) Bulk δ^13^C composition of carbonate veins (circle: dolomite, triangle: magnesite, and square: calcite) in New Caledonia and Oman (2σ 0.06‰). Red circles: this study (See Fig. S11 for the location of the micro-drilling). Black symbols: literature data^21,31,41–43,56^. The gray band corresponds to the δ^13^C composition of dolomite-calcite veins in the Mid-Atlantic Ridge where CH_4_ production has been proposed^11^; (**b**) magnetite-dolomite co-crystallization temperatures estimated from equilibrium oxygen fractionation between the two minerals; (**c**) simulated fluid composition in equilibrium with GR2H vein mineralization at 95 °C and after cooling down to 30 °C. The Ca content is increased stepwise and [CO_2_] corresponds to dolomite saturation at each step. The chemistry of the alkaline source water at the *La rivière des Kaoris*^37^ which is not contaminated by seawater, is plotted for comparison.

In situ δ^18^O V-SMOW and δ^13^C V-PDB data were collected using SIMS in three zones (Fig. 3 and Fig. S10) of the GR2H vein network. The δ^18^O data range from − 10.4 to − 14.2‰ and 19.6 to 21.8‰ for magnetite and dolomite, respectively (Supplementary Table S3, Fig. 3); δ^18^O variation within a single grain is below 1‰. In situ δ^13^C data for dolomite range from 7.1 to 17.3‰ (Supplementary Table S4, Figs. 3 and 4a). Additional δ^18^O and δ^13^C data were obtained by IRMS using the micro-bulk method (see “Methods”) on dolomite powder drilled from the vein samples (Fig. S11). Micro-bulk δ^18^O and δ^13^C data range from 21.3 to 22.6‰ and from 6.3 to 11.5‰ (Supplementary Table S5), respectively, in good agreement with the in situ data.

### Isotopic equilibrium temperature and fluid chemistry

Isotopic equilibrium temperatures were retrieved from the temperature dependency of the magnetite—dolomite oxygen fractionation factor (Supplementary Item 1). Temperatures comprised between 94 and 117 °C were obtained, which cluster at 97 ± 5 °C (Fig. 4b, Supplementary Table S6) and will be rounded to 95 °C in the following. It is interpreted as the precipitation temperature of magnetite and dolomite in the veinlets. The δ^18^O of the solution from which they co-precipitated is expected to range from 1.2 to 3.5‰ as calculated with the dolomite–water oxygen isotope fractionation factor^44^.

The composition and pH of the fluid from which the vein minerals precipitated were modeled at 95 °C using PHREEQC^45^ (see “Methods”). This simulation was also aimed at testing the stability of the vein mineral assemblage. One mole of olivine ((Mg_0.9_Fe_0.1_)_2_SiO_4_) was reacted with one liter of water, corresponding to a water-to-rock mass ratio of ca. 7, with the constraint of dolomite saturation and calcite/magnesite undersaturation. Serpentine, chrysotile, magnetite, Fe-brucite, and H_2_ (32 × 10^–2^ mol/L) were produced along with dolomite, in good agreement with the observed GR2H veinlet mineralogy. The simulation did not yield stable pyroaurite, which is consistent with the notion that pyroaurite is a late alteration product of brucite. The modeled equilibrium fluid at 95 °C (Solution 1) has a pH close to 8.5–9 and a [Ca] comprised between 0.02 × 10^–3^ and 0.2 × 10^–3^ mol/L (Fig. 4c). Below and above that [Ca] range, magnesite and calcite saturation are achieved, respectively. The cooling of Solution 1 from 95 °C down to 30 °C was then processed in order to simulate the fluid composition (Solution 2) when emitted at the surface in a 30 °C hydrothermal spring. Solution 1 was merely run at 30 °C by allowing minerals to precipitate. Serpentine + dolomite and serpentine + calcite were found to precipitate at lower and higher Ca concentrations, respectively. The simulated composition range of Solution 1 (95 °C) and Solution 2 (30 °C) is depicted in Fig. 4c and compared to alkaline waters issued at *La rivière des Kaoris* spring.

## Discussion

**Figure 5:**
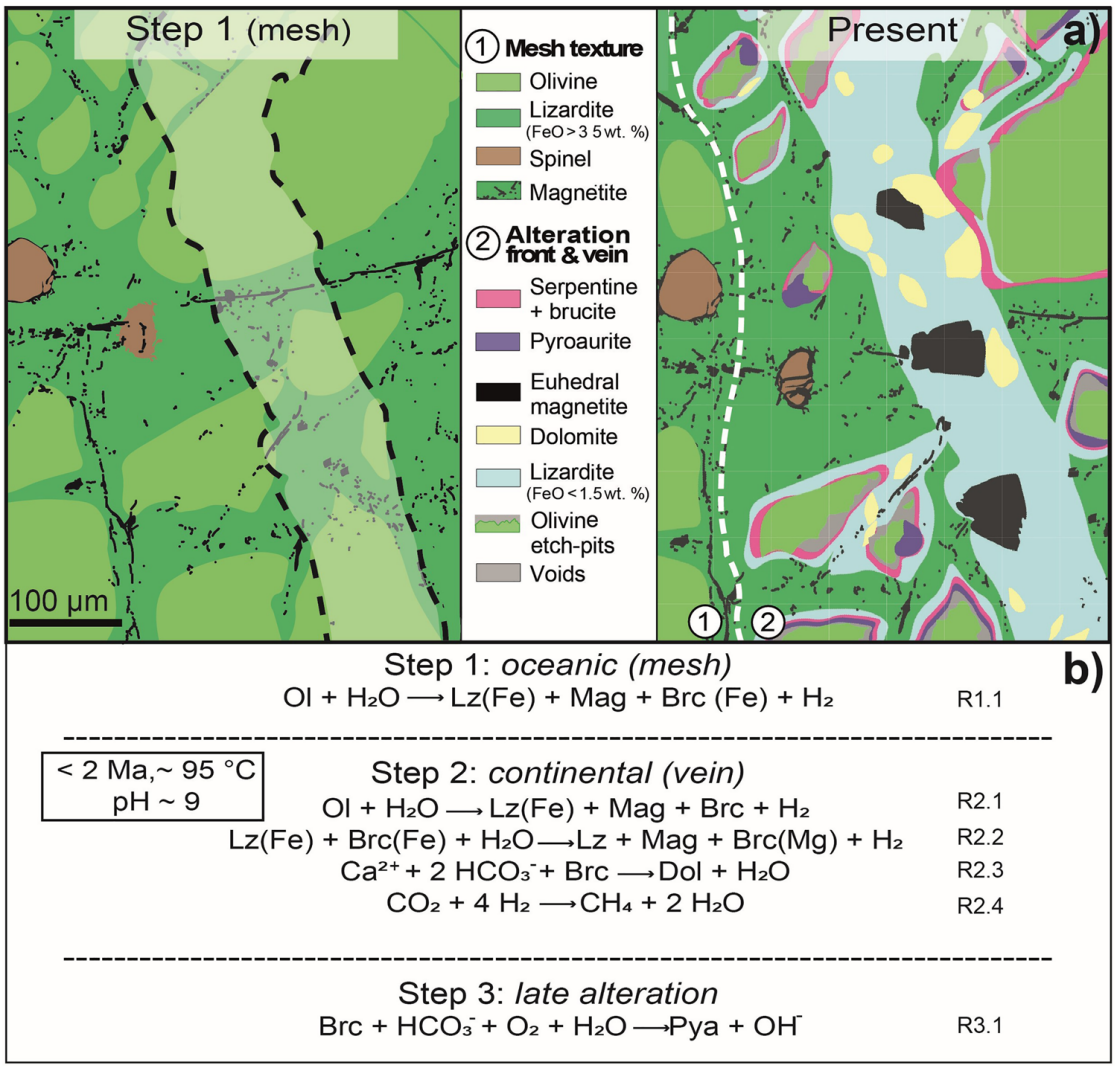
Summary of the water–rock interaction events recorded by the GR2H sample. (**a**) Schematic diagram based on the SEM image displayed in Fig. 2c. Three steps can be distinguished based on mineralogical and textural features. Magnetite–dolomite–lizardite vein (Step 2, right panel) crosscut and thus postdate the mesh texture (Step 1, left panel) highlighted by the alignment of small magnetite grains (black). Pervasive olivine alteration in Step 2 occurs within an alteration front of a few hundreds of µm, and gradually increases towards the vein. Olivine embedded in the vein material displays pronounced alteration features (etch pits). Fe-poor lizardite and dolomite are also located in the vein as well as in the alteration front. A late alteration stage (Step 3) led to the formation of pyroaurite, which involved CO_2_-rich and oxygenated waters. (**b**) Main chemical reactions associated with each of the three steps described in (**a**), deduced from mineral textures, isotopic data and thermochemical modeling. Lizardite and brucite with the highest Fe content are named Lz(Fe) and Brc(Fe) respectively, whereas Brc(Mg) means Fe-poor brucite.

The GR2H sample recorded different steps of fluid-rock interaction. The earliest step that is preserved (Fig. 5, Step 1) corresponds to oceanic serpentinization showing the typical mesh texture. Residual olivine from Step 1 is further altered within and next to dolomite-bearing mm-sized veins (Fig. 5, Step 2, R2.1). The overall chemical reaction (Fig. 5b, Step 2) accounting for the observed mineralization involved both hydration (Fig. 5b, R2.1 and R2.2) and carbonation (Fig. 5b, R2.3). The olivine dissolution features, more pronounced in the vicinity of the vein wall, as well as the textural relationships between dolomite, Fe-poor serpentine, and magnetite (Fig. 5) suggest the following overall reaction (Step 2):

$$\begin{gathered} \left( {{\text{Mg}}_{{0.{9}}} {\text{Fe}}_{{0.{1}}} } \right)_{{2}} {\text{SiO}}_{{4}} + {\text{ CO}}_{{{2},{\text{aq}}}} + {\text{ Ca}}_{{,{\text{aq}}}} + {\text{ H}}_{{2}} {\text{O }} = > {\text{ Mg}}_{{3}} {\text{Si}}_{{2}} {\text{O}}_{{5}} \left( {{\text{OH}}} \right)_{{4}} + {\text{ CaMg}}\left( {{\text{CO}}_{{3}} } \right)_{{2}} + {\text{ Fe}}_{{3}} {\text{O}}_{{4}} + \, \left( {{\text{Mg}},{\text{Fe}}} \right)\left( {{\text{OH}}} \right)_{{2}} + {\text{H}}_{{2}} \hfill \\ olivine + \, aqueous \, species = > \, Fe - poor \, lizardite \, + \, dolomite \, + \, magnetite \, + Fe - brucite \, + \, H_{2} \left( {R1} \right) \hfill \\ \end{gathered}$$

Finally, a later stage (Fig. 5b, Step 3) led to the oxidation and the carbonation of Fe-brucite into pyroaurite (Fig. 5b, R3.1). Pyroaurite is likely a late product arising from the oxidation/carbonation of former ferroan brucite^25^. This assumption is supported by thermochemical modeling and by electron microprobe data which show that the Mg-content of pyroaurite (Mg# ~ 0.2) is similar to that expected for brucite usually found in ophiolites^46^.

Thermochemical modeling shows that the serpentine—dolomite—magnetite—Fe-brucite assemblage, has a stability field at 95 °C in the [CO_2,aq_]–[Ca^2+^] space in equilibrium (e.g., for [CO_2,aq_] and [Ca^2+^] ~ 10^–4^ mol/L.) with an alkaline aqueous solution (8 < pH < 9). Dolomite is a common alteration product of ultramafic rocks^47–50^. The LT serpentine (Step 2) described here is noticeably Fe-poor (Mg# = 0.98). Such low Fe content is expected for serpentine minerals produced at the later stages of serpentinization, along with magnetite and H_2_^51^. Iron depletion in serpentine found in successive vein generations has already been described in a New Caledonia dunite and interpreted as the transition from rock-dominated to fluid-dominated systems subjected to fluid infiltration^46^. The authors proposed that the infiltrating oxidizing fluid is reduced by the extraction of iron from early serpentine and brucite to form magnetite and H_2_ (Fig. 5b, R2.2). This is to say that the overall R1 reaction may have proceeded with an intermediate step of Fe-rich serpentine production (Fig. 5b, R2.1), i.e. in a rock-dominated system, followed by Fe-poor serpentine and magnetite formation in a fluid-dominated system^46^. The presence of residual olivine in the vicinity of the studied vein (< 50 µm) ensured that both low SiO_2_ activity and low *f*O_2_ prevailed during this latter mineralization stage^51^ (Step 2). Therefore, although no fluid inclusion could be found in these veinlets to support the presence of H_2_, mineral compositions and textures strongly suggest a late serpentinization stage with H_2_ production as predicted by thermochemical modeling of R1 at 95 °C. This modeling, however, is partly hampered by the lack of thermodynamic data accounting for iron incorporation into serpentine minerals. Serpentine has been computed as pure Mg end-member. The magnetite and H_2_ amounts calculated with a pure Mg end-member are likely to be overestimated due to the iron affinity with respect to serpentine at T < 150 °C^52^.

Both the Ca content and pH of the percolating aqueous fluid inferred from thermochemical modeling at 95 °C are consistent with the Type-II Ca–OH waters typically encountered during alteration of partially serpentinized peridotites in the deeper levels of ophiolites. The crystallization of the dolomite-serpentine-magnetite assemblage can be seen as a mean to shift from a Type I to a Type II water composition in a fluid-dominated system.

Multiple grain dating of magnetite from GR2H dolomite-bearing veins yielded Quaternary ages. Helium is retained within the magnetite crystal structure at the temperature (ca. 95 °C) of GR2H magnetite formation^53^. In this case, MgHe system is a chronometer and allows to date magnetite crystallization and thus Step 2 (Fig. 5). Magnetite crystallization may have proceeded according to successive growth episodes (see growth zones Figs. S3 and S4) which cannot be dated individually but which yielded an average Quaternary age. In addition, young MgHe ages (Quaternary) were also obtained for GP magnetite, implying that Quaternary fluid–rock interactions have affected the two localities (GR2H and GP). The Quaternary MgHe ages were rather unexpected. Indeed, based on the syn-kinematic character inferred for fluid infiltration and lateritization, Oligocene ages related to peridotite nappe emplacement were expected. Actually, paleomagnetic data^33^ show that lateritization of the southern part of the New Caledonia ophiolite began during the late Oligocene and proceeded through Pliocene–Quaternary times. Geomorphological data indicate post-obduction tectonic activity with an uplift component associated with erosion^33^ that may have reactivated fluid conduits and low-temperature serpentinization/carbonation up to very recent times.

Without the constraint of the MgHe dates, the meteoric origin of the fluid would have been difficult to assess from its calculated equilibrium δ^18^O composition (1.2–3.5‰) alone. Typical δ^18^O for meteoric and seawater range from slightly negative^54^ to near zero, respectively. The serpentine—water fractionation factor amounts to ca. 7‰ at 95 °C according to the calibration by Wenner and Taylor^15^. Therefore, a way for a fluid to reach 1.2–3.5‰ would be to equilibrate with abundant serpentine (i.e. low water to rock ratio) having δ^18^O in the 8.2–10.5‰ range. However, in the New Caledonia ophiolite, apart from rare examples of serpentinites with δ^18^O above 8‰, most of them cluster around 5.5‰^32^. The way the percolating fluid has acquired its oxygen isotopic composition remains to be determined.

Based on the crystallization temperature (~ 95 °C) derived from oxygen isotopes, it can however be concluded that a large-scale drainage system was active about 2 Ma ago, which involved the percolation of meteoric water through the partly serpentinized ophiolite at depth. Note that fluids with temperatures of up to 80–95 °C were also reported in the Koniambo massif in the northern part of New Caledonia based on the oxygen isotopic composition of quartz veins^55^. The corresponding heat source remains unclear.

The modeled pH and composition (dissolved Ca, Fig. 4c) of water in equilibrium with the GR2H mineralization and then cooled down to 30 °C approach those reported at *La rivière des Kaoris* spring^37^. The slight difference in modeled and measured pH and [Ca] might be accounted for by uncertainty in the thermochemical data used for the modeling as well as the departure from equilibrium (mineral supersaturation) encountered for *La rivière des Kaoris* water^37^. A possible connection between the mineralizing fluid described here and (hyper)alkaline sources such as those venting H_2_ (and CH_4_) today in the Massif du Sud remains plausible from a geochemical point of view. In the Samail Oman Ophiolite, late Ca-carbonate vein/veinlet networks prevail in highly serpentinized peridotites near low-temperature alkaline springs emanating from the peridotite^56^. This observation indicates that alkaline springs are fed by a fracture network comprising veins that promote chemical exchanges between the peridotite and the percolating aqueous fluid. In other words, the magnetite–dolomite–serpentine mineralization can be seen as part of the plumbing system that fed hyperalkaline sources. The small size of the veins along with pervasive fluid infiltration normal to the veins (Fig. 5a), imply a high exchange surface per percolating fluid unit for LT serpentinization and H_2_ production.

Another strong argument in favor of a connection to current alkaline sources is the highly positive δ^13^C value (> 6.3‰) of dolomite in GR2H (Fig. 4a, Tables S4 and S5) which can be interpreted as the result of CH_4_ production. Indeed, δ^13^C values of 7.2–8.7‰ were measured in calcite–dolomite veins in Mid Atlantic Ridge 15°N^11^ and were attributed to the effect of methanogenesis (Fig. 4a). The formation of methane with highly negative δ^13^C values would result in the shift to positive δ^13^C values of the remaining aqueous CO_2_^57,58^ and thus explain why the δ^13^C values of dolomite, which incorporates this aqueous CO_2_, is so exceptionally high.

In summary, the multiple approach followed here strongly supports the hypothesis that the studied magnetite–serpentine–dolomite veinlets are the products of LT serpentinization and carbonation by an aqueous fluid of meteoric origin which led to the production of H_2_. Further interaction of H_2_ with aqueous CO_2_ produced CH_4_ (Fig. 5b, R2.4) through either a Sabatier reaction or by microbial hydrogenotrophic methanogenesis^38^.

## Conclusions

Whereas serpentine + magnetite veins on the one hand and carbonate veins on the other are commonly described in altered ophiolites, the vein mineralization studied here includes both serpentinization products and carbonates. The corresponding vein mineral assemblage, which formed at 95 °C less than 2 Ma ago, illustrates the interplay between serpentinization and carbonation, at the microscale, for the formation of alkaline fluids in the New Caledonia ophiolite. Indeed, the serpentine–magnetite–dolomite assemblage fits perfectly with the expected mineralogy for the production of a Ca-OH (Type-II) waters from Mg^2+^- and HCO_3_^−^-rich water (Type-I). Low-T serpentinization proceeds at ca. 95 °C and consists of the reaction of olivine and mesh serpentine (and possibly Fe-brucite) with a CO_2_-bearing fluid to form dolomite + Fe-poor lizardite + magnetite + Fe-brucite + H_2_ in a second alteration step. In addition, based on carbon isotopic compositions of dolomite, it is expected that part of the aqueous CO_2_ has reacted with H_2_ to produce methane.

On a methodological viewpoint, the MgHe method was already used to date LT magnetite crystals (< 60 °C) in serpentine-free calcite veins from the Samail ophiolite (Oman)^59^. It is a suitable tool to put time constraints on fluid–ultramafic rock interactions at low temperature using magnetite from veins (high water/rock ratio).

The present approach, which combines the MgHe method with mineralogical and geochemical characterization (textures, mineral chemistry, and isotopic data), prompts a regional-scale study to constrain fluid pathways and possibly characterize the subsurface plumbing system and catchment volume of former (Quaternary) alkaline springs.

## Methods

### Petrography and mineral characterization

The investigated samples were selected for the presence of magnetite with the largest sizes for MgHe dating. Magnetite crystals above 400 µm in diameter were found in millimeter-sized veins, sampled at the Georges Pile (GP) and GR2H sites. Sample mineralogy has been characterized on thin sections and polished rock fragments. In order to retrieve serpentinization degree, olivine and spinel areas were counted manually two times independently on assembled microscope (Olympus Cover-018 BH2) images covering 4 × 4 mm (100 points, reflected light). Textural relationships (e.g., Fig. 2) among mineral phases of the GR2H sample were characterized using scanning electron microscopy (Vega3 Tescan, ISTerre) equipped with a 30 mm^2^ SDD X-ray detector for semi-quantitative analysis. Serpentine and carbonate mineralogy were determined by Raman micro-spectrometry using a LabRAM Soleil Horiba Scientific (ISTerre, Grenoble) with a 532 nm length wave laser at 6 mW. A silicate glass was used for calibration. Raman spectra were collected using an acquisition time of 5 s with 10 accumulations per spectrum. Grating and pinhole were 600 grooves per mm and 100 µm, respectively. Raman spectra of serpentine (lizardite), dolomite and pyroaurite from the GR2H sample are displayed in Fig. S7. Quantitative mineral analyses (Supplementary Table S1) and elemental X-rays maps (Figs. S3 and S4) were collected with the electron microprobe (JEOL FEG JXA-iHP200F at ISTerre, Grenoble). Acceleration voltage was 15 keV and beam current was 100 nA for magnetite and dolomite point analyses and 6 nA for serpentine and brucite/pyroaurite point analyses. The counting time was 120 s per analysis. For the electron probe x-ray maps, the beam current was increased to 300 nA and 100 nm steps were used with 60 s counting time. Standardization was made using certified minerals, pure metals, and synthetic oxides: wollastonite (Si et Ca), rutile (Ti), NiO (Ni), rhodonite (Mn), Cr_2_O_3_ (Cr), Al_2_O_3_ (Al), periclase (Mg), CoO (Co), sphalerite (Zn), magnetite (Fe), cancrinite (Cl), SrSO_4_ (Sr).

### Magnetite (U-Th)/He geochronology

The (U-Th)/He method is based on the production and accumulation of He in the crystal structure due to the alpha decay of U, Th and Sm atoms. He accumulation within the crystal structure will depend on He diffusion coefficient which is temperature dependent. In the case of magnetite, He is well retained for T < 150 °C^53^. Euhedral magnetite crystals with a sphere radius > 400 µm were extracted from carbonate veins in the GR2H samples and from serpentine veins in the GP samples with a blade. All crystals were hand-rubbed on a P600 sandpaper to physically remove crystal edges and goethite alteration rim in the case of GP magnetite. About thirty crystals from Georges Pile and GR2H samples were mounted for CTscan analysis^60^ using an EASYTOM XL nanofocus tomograph at CMTC (Grenoble University, France, Fig. S9) with a beam current of 76 nA and a voltage of 20 V. High-resolution CDD camera (40 pl/mm) was used with a matrix of 4000 × 2064 pixels and a pixel size of 5.9 μm. Crystals containing the fewest or no mineral inclusions were selected and 3 to 11 crystals were wrapped in a niobium foil into five aliquots (two from GR2H and three from GP; more detail in Supplementary Table S2). They were then degassed under vacuum with an ytterbium doped diode laser for 30 min at a temperature of 1000–1200 °C, spiked with a known amount of ^3^He. Extracted gas was purified and analyzed for ^4^He with an error estimated at 2% using the Pfeiffer® Quad-line Prisma QMG 100 at the GEOPS (Paris—Saclay University, France) following the protocol described in Gautheron et al.^61^.

Aliquots from GR2H was dissolved in 2 mL Savilex® Teflon microbombs with 1.5 mL aqua regia (one volume of 10.5 N HCl and three volumes of 18N HNO_3_), 0.5 mL of 29N HF, and 2 drops of concentrated HClO_4_. A spike (10 µL) composed of ^235^U and ^232^Th and ^149^Sm was added to the solution. The Savilex® Teflon microbombs were placed on a hot plate at 130 °C for 24 h until complete digestion. Aqua regia and HF were first evaporated at 130 °C then HClO_4_ was evaporated at 180 °C. The resulting solid residue was dissolved with 0.5 mL of 1N HNO_3_ on a hot plate at 100 °C. After digestion, the solutions were diluted with 10 mL of 0.5N HNO_3_ to Fe concentrations (< 1500 µg/g) appropriate for HR-ICP-MS analysis.

Aliquots from Georges Pile were dissolved with 50 µL of 5N HNO_3_ and 750 µL of HF with 100 mL of 5N HNO_3_ containing known amounts of ^235^U, ^232^Th, and ^149^Sm into a Savillex vial on a hot plate at 150 °C during 24 h. Acids were evaporated at 100 °C, then 750 µL of concentrated HCl are added and solutions were heated at 150 °C during 24 h. The HCl was evaporated and then, 400 µL of 7N HNO_3_ were added at 150 °C. Dissolution was completed in 2 h. Finally, the solutions were diluted to reach 10 ppm of Fe.

U, Th, and Sm in the solutions were analyzed using ICP-MS Thermo® Element XR (IUEM, Brest University, France) for GR2H aliquots. ICP-MS Thermo® Element XR (GEOPS, Paris—Saclay University, France) was used for Georges Pile aliquots using the isotope dilution method^62^.

### Oxygen and carbon stable isotope measurement

In situ oxygen (δ^18^O V-SMOW) and carbon (δ^13^C V-PDB) isotope data were collected on magnetite and dolomite using the IMS 1270 CAMECA ionic probe in a multi-collection configuration at CRPG (Nancy, France). Measurements were conducted with a 2 nA Cs + primary beam. The transmission is 80 and the mass resolution is 5000. The detectors used to analyze ^16^O and ^17^O are L2(Fc) and H1 (Fc) respectively. The pre-sputtering is 90 s with a raster of 30 µm, and then, 20 µm for the measurement. For the analysis of δ^13^C, the detectors used are C (Fc) for ^12^C measurement and H_2_ (EM) for ^13^C measurement. The pre-sputtering is 120 s with a raster of 25 µm and then 20 µm during the measurement.

The studied samples are three polished fragments (5 × 5 × 0.5 mm) of GR2H dolomite veins (P1S2, P1S4, P4S4; see Fig. 3 and Fig. S10) which were pressed into indium within a 25.4 mm diameter aluminum ring^63^. In situ analyses of dolomite were calibrated with an in-house standard with a δ^18^O V-SMOW isotopic composition of 20.04‰ and δ^13^C V-PDB composition of 3.56‰. An in-house standard (δ^18^O = 1.42‰) was used to calibrate the δ^18^O V-SMOW of magnetite. Before and after each session, the standard was measured six times to obtain the instrumental drift. The difference between the reference isotopic composition and the isotopic composition measured on the standard during the session is corrected on samples as well as the instrumental drift.

Micro-bulk δ^18^O (V-SMOW) and δ^13^C (V-PDB) analyses on dolomite were performed using an auto-sampler Gasbench coupled to a Thermo Scientific MAT253 isotope ratio mass spectrometer (IRMS) at the CRPG (Nancy, France). Between 0.19 at 0.37 mg of vein material was extracted from the GR2H sample (P2S3, P3S3, P3S4; see Fig. S11) using a micro-drill. The procedural preparation and the analysis method presented in Fallick et al.^64^ were followed.

All sample measurements were calibrated to the in-house reference (MCt: Merck synthetic calcite, δ^13^C = − 8.63‰ V-PDB; δ^18^O = − 17.90‰ V-PDB) calibrated on the international standards IAEA CO-1, IAEA CO-8 and NBS 19. Reproducibility was better than 0.1‰ and 0.05‰ for δ^18^O and δ^13^C, respectively.

### Computation of the aqueous fluid composition using PHREEQC

The composition of the aqueous solution in equilibrium with vein minerals has been modeled at 95 °C using PhreeqC—Version 3^45^ and the LLNL.dat database. Fe(OH)_2_ data^65^ were implemented in the database (Suppl. Data). The Fe(OH)_2_–Mg(OH)_2_ solid-solution was assumed to be ideal. Pyroaurite thermochemical data^66^ were implemented in PhreeqC (Suppl. Data) and were then tested against the dissolution dataset that was used to retrieve them. Due to the lack of data on CH_4_ formation kinetics in the CO_2_-H_2_-H_2_O system, calculations with olivine as starting material to evaluate the H_2_ production in the presence of dolomite, were performed without allowing for CH_4_, CO or C formation.

### Supplementary Information

Supplementary Information 1.

Supplementary Information 2.

## Data Availability

All data generated or analyzed during this study are included in this published article and its supplementary information files.
