# Supplementary material for: Quaternary low-temperature serpentinization and carbonation in the New Caledonia ophiolite
Source: Sci Rep. 2023 Nov 8;13:19413. doi: 10.1038/s41598-023-46691-y (PMC10632469; doi:10.1038/s41598-023-46691-y)
Supplement: Supplementary file 2 — Supplementary Information 2. [file 41598_2023_46691_MOESM2_ESM.docx]

**Supplementary information**

**Quaternary low-temperature serpentinization and carbonation in the New-Caledonia ophiolite**

By M. Corre, F. Brunet, S. Schwartz, C. Gautheron, A. Agranier and S. Lesimple

**The supplementary information includes:**

- **Item 1: Calculation of dolomite-magnetite isotopic temperature of crystallization**
- **Supplementary figures**
- **Supplementary tables**
- **Thermodynamic data for the phases added to the LLNL PHREEQC database**
- **References cited**

**Calculation of dolomite-magnetite isotopic temperature of crystallization**

Since the textural relationship between magnetite and dolomite indicates that they have co-crystallized, the temperature retrieved from oxygen isotope fractionation between the two minerals was interpreted as their crystallization temperature. The δ^18^O composition of magnetite and neighbor dolomite that was measured *in situ* on polished sample fragments (Fig. 3 and S7) were used to calculate their precipitation temperature (Table S6) from the temperature dependency of the magnetite – dolomite fractionation factor^1^:

$1000ln\alpha_{Mag-Dol}=A\times\left( \frac{{10}^{6}}{T^{2}} \right)+B\times\left( \frac{{10}^{3}}{T} \right)+C$ (*E1*)

where 1000ln*∝_Mag-Dol_* is the oxygen isotope fractionation factor for the magnetite -dolomite pair in ‰; T is the equilibrium temperature in K and A, B and C are constants.

The temperature dependency of 1000ln*∝_Mag-Dol_* has been derived from magnetite and dolomite fractionation data from Chacko et al. (2001)^2^ and Zheng (2011)^3^, respectively. Temperature data are given as median value +/- 2 MAD (Median Absolute Deviation).

**Supplementary figures**

All mineral abbreviations are from Warr (2021)^4^.


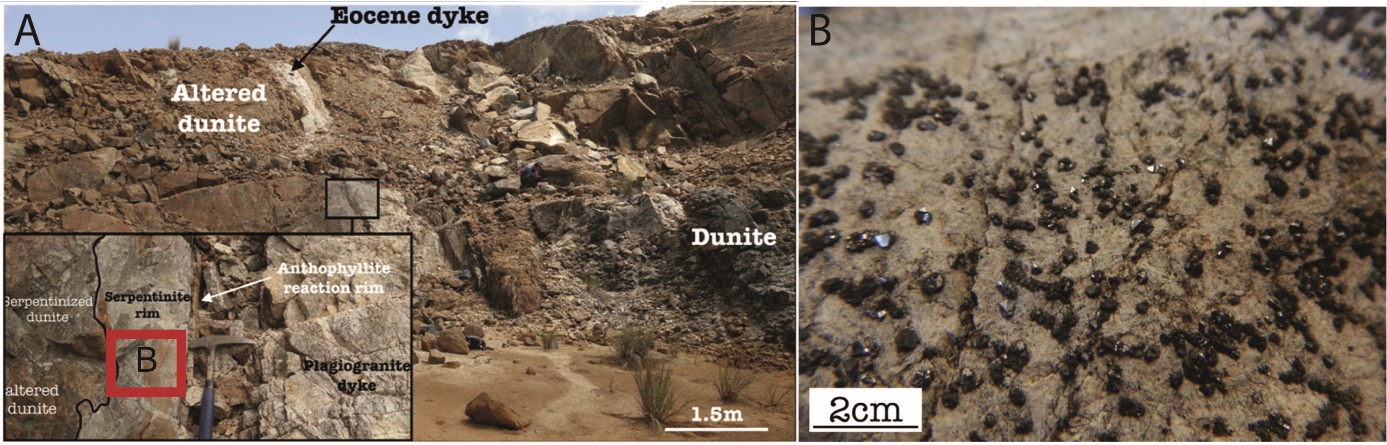


**Figure S1.** Environment of the Georges Pile samples and magnetite habitus. A) Zone of preferential aqueous fluids circulation (ca. 30 cm thickness) at the interface between a plagio-granite dyke dated from 27 to 24 Ma^5^ and the host serpentinized dunite: B) Habitus of the magnetite dated with the MgHe method, the corresponding sample was collected at the serpentinite rim (red square in A).


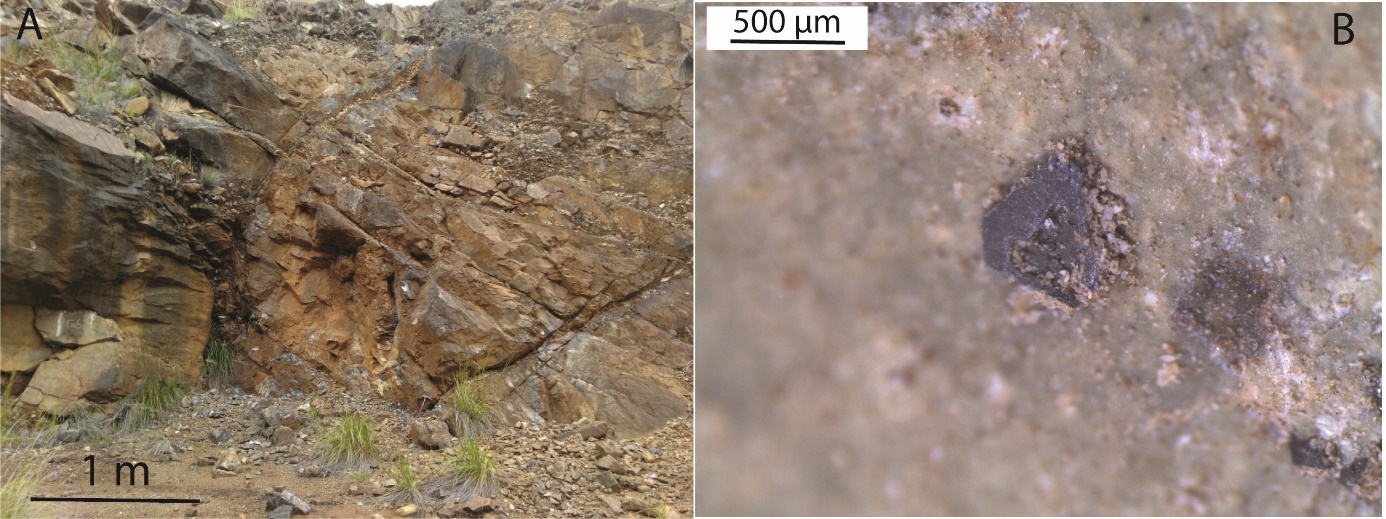
**Figure S2.** Environment of the GR2H samples and magnetite habitus. A) Outcrop; B) Euhedral magnetite with dolomite in a vein.


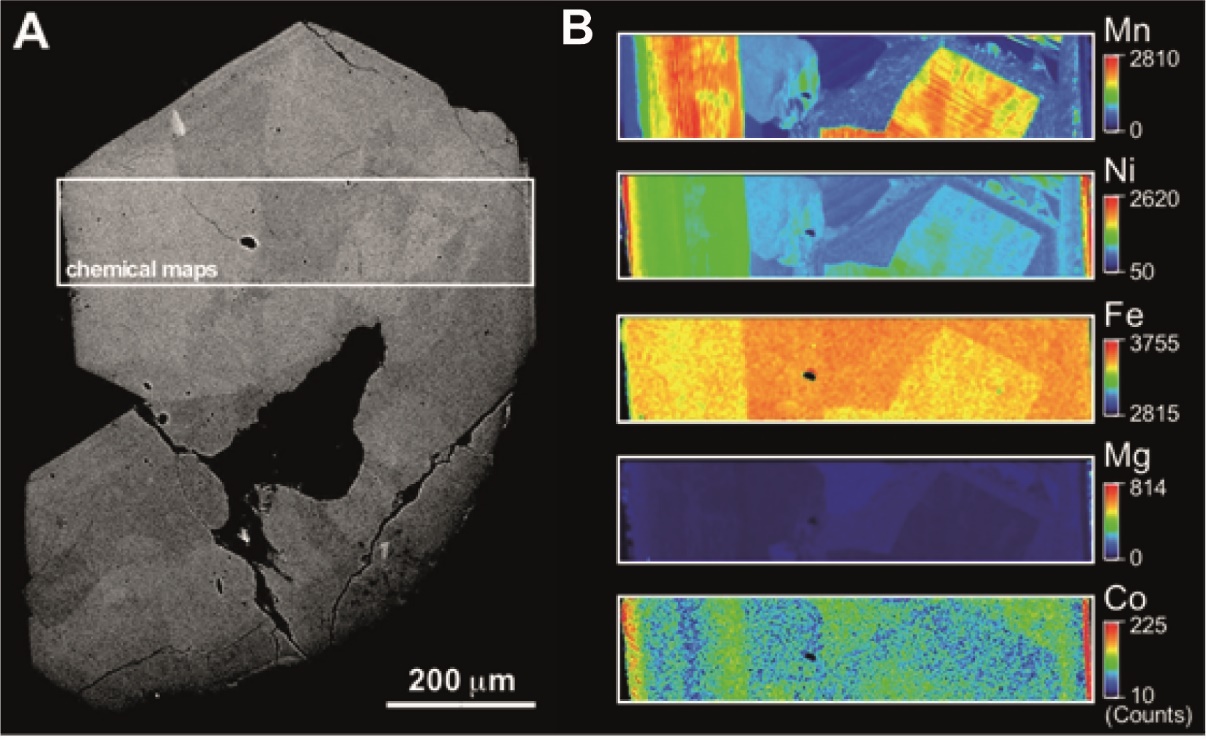


**Figure S3.** Chemical zoning in magnetite from Georges Pile. A) Back-scattered electron image of GP magnetite with the location of the EPMA X-ray maps displayed in B) where chemical zoning (Mn, Ni, Fe, Mg, and Co) is emphasized.


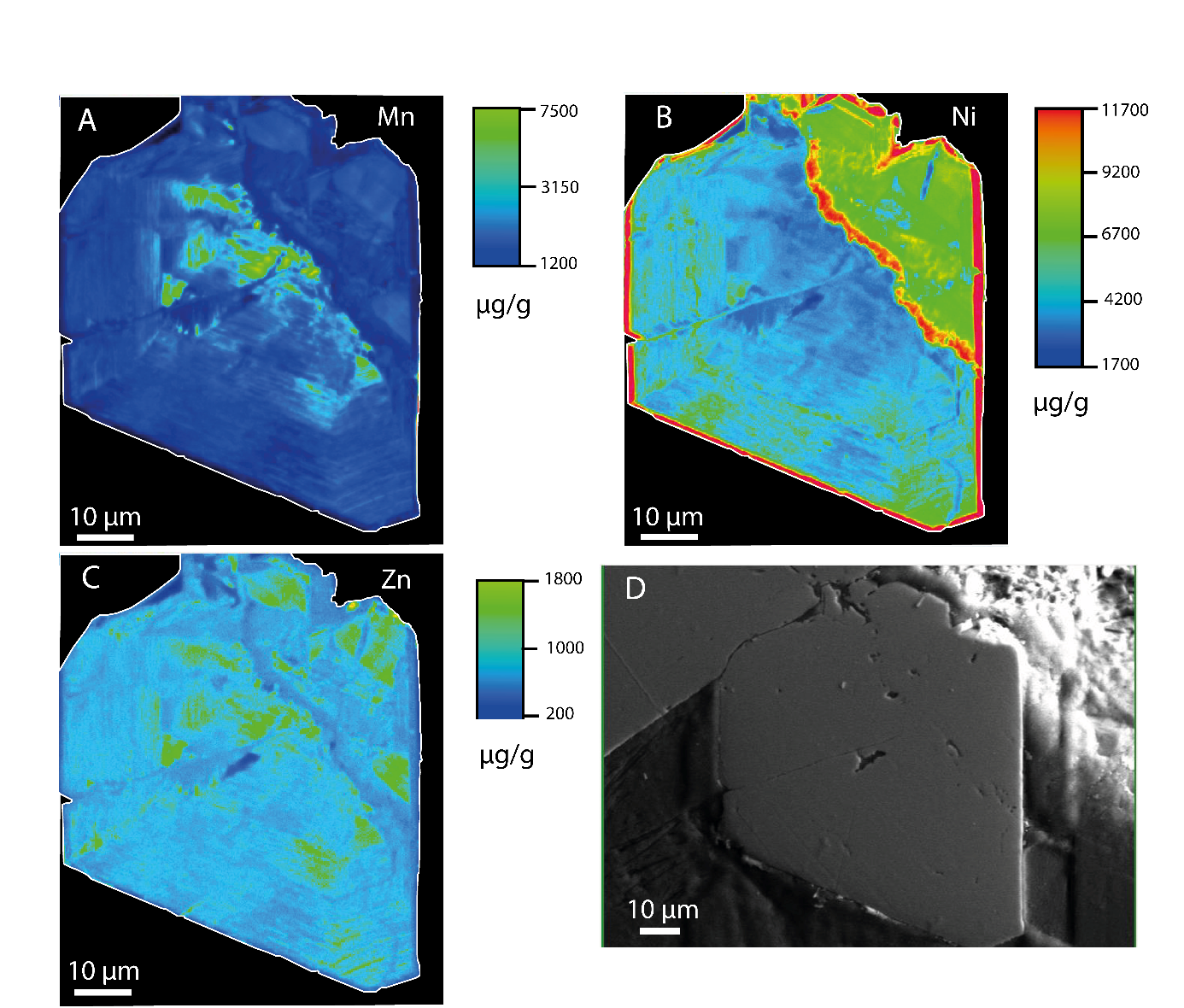
**Figure S4.** Chemical zoning in magnetite from GR2H. A), B), and C) are EPMA X-ray maps for Mn, Ni, and Zn, respectively; D) Corresponding back-scattered electron image. The magnetite grain is enriched in Ni close to the veinlet edge from which it started to grow.


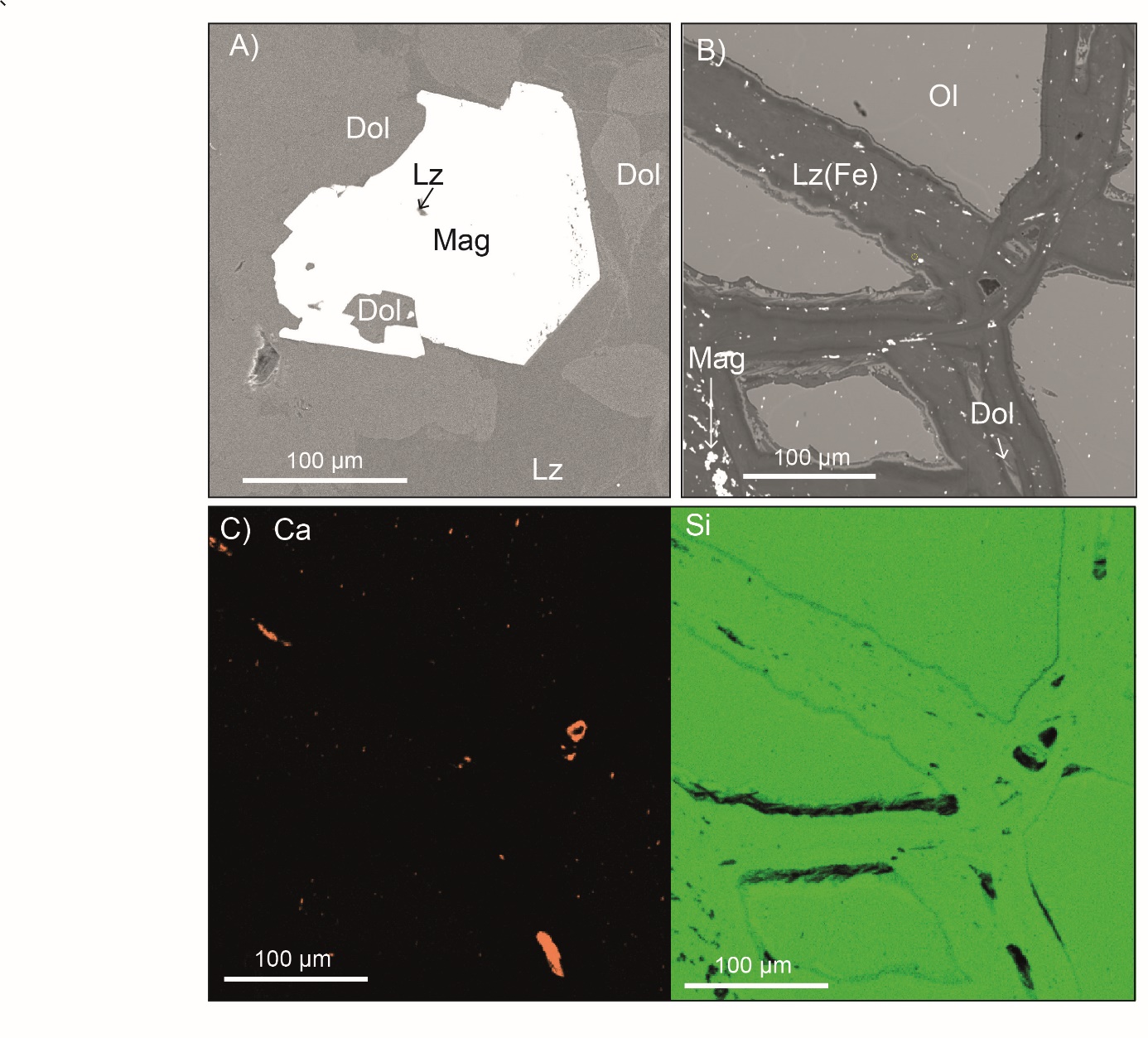


**Figure S5**. **A)** SEM image (BSE mode) showing the textural relationship between euhedral magnetite and dolomite within a GR2H veinlet. Dolomite either crystallized in sharp contact with magnetite or was even enclosed into magnetite (in a 2D-section). Note that lizardite is also included in magnetite. **B) and C)** Detail of the serpentine/magnetite mesh texture in the vicinity of the dolomite vein where dolomite grains crystallize after the mesh. Dolomite is located on the x-ray maps (SEM) in Si-free zones containing Ca.


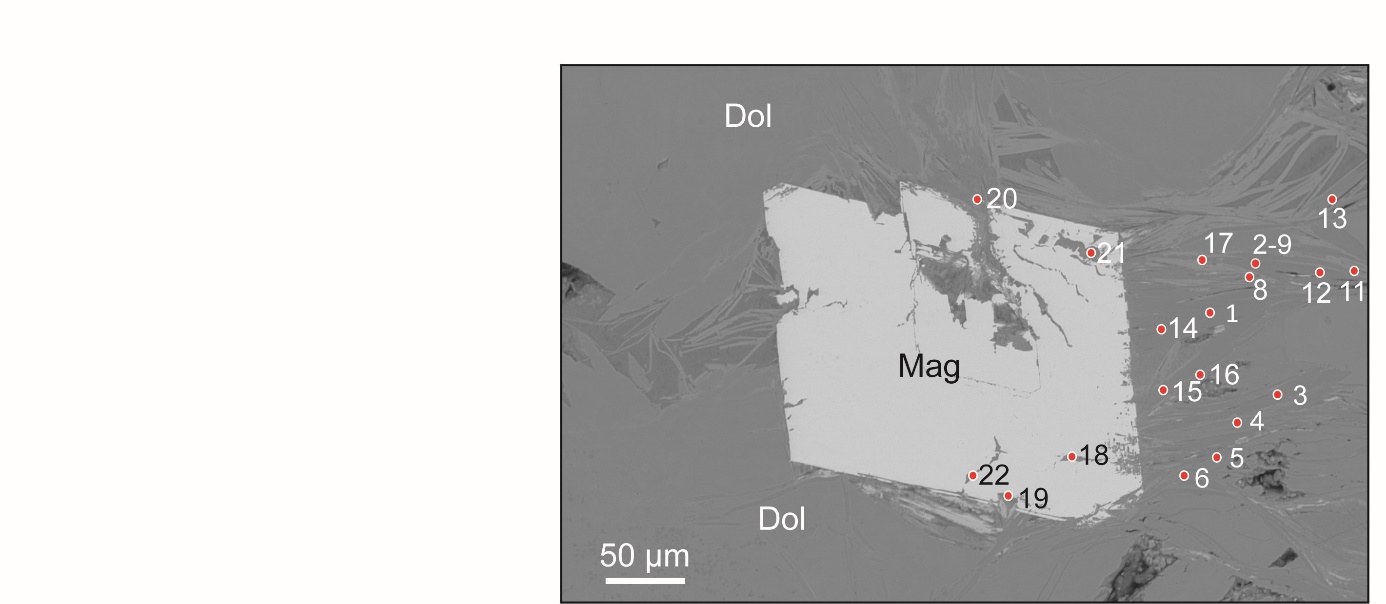


**Figure S6.** SEM image showing the veinlet mineralization at the vein wall – Dol: dolomite and Mag: magnetite. Phases identification was achieved using micro-Raman (Fig. S7): pyroaurite (1, 2, 3, 8, 9, 12, 18, 19, and 22), dolomite (5 and 6), lizardite (20 and 21), pyroaurite-lizardite (11, 13, 14, 15, and 17), and pyroaurite-lizardite-dolomite intergrowth (4 and 16).

**
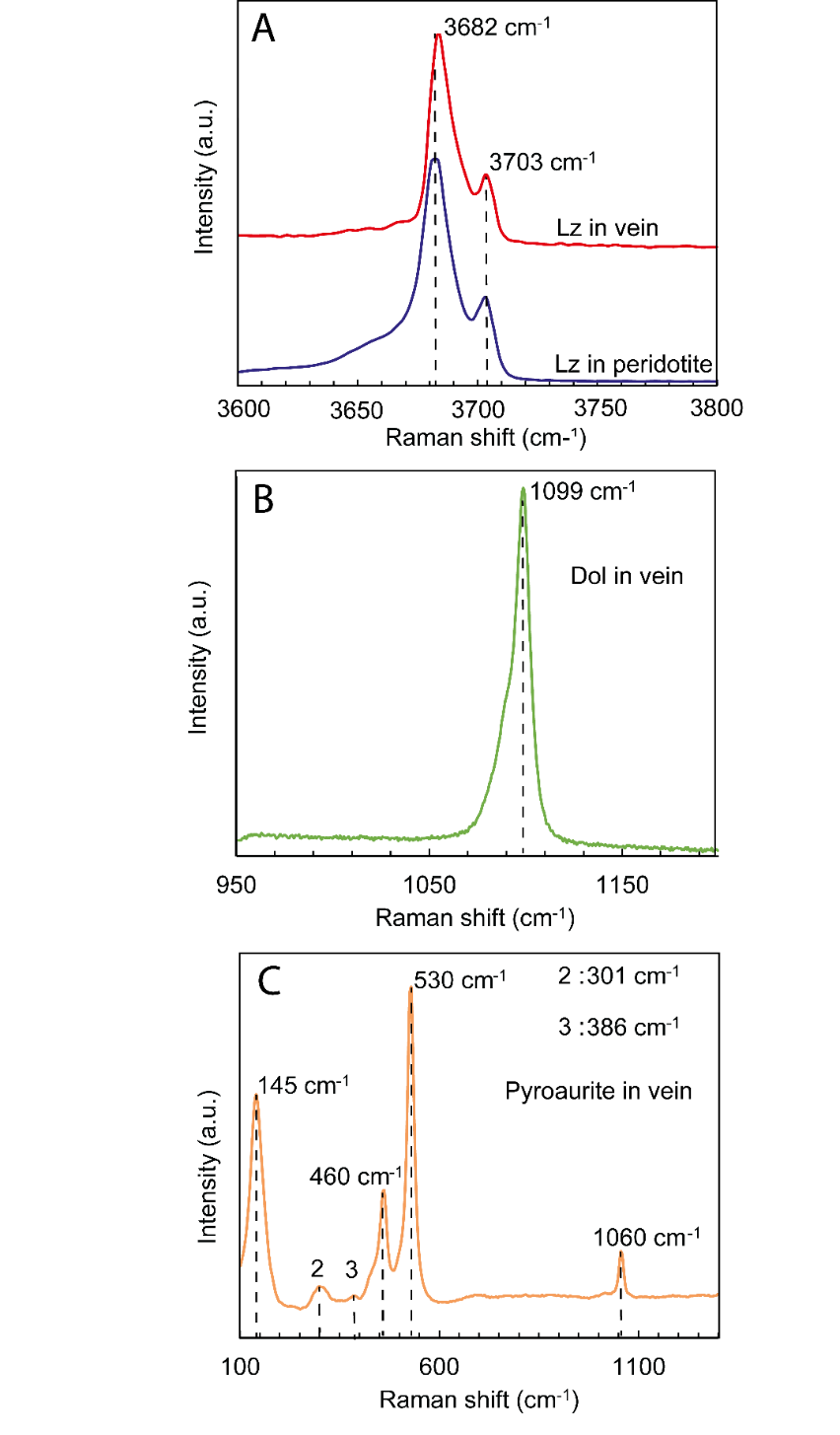
**

**Figure S7.** Raman spectra of serpentine, dolomite and pyroaurite from GR2H. A) Representative spectrum of serpentine in both veinlets and host peridotite, identified as lizardite (characteristic bands^6^ : 3680 cm^-1^, 3703 cm^-1^). B) Representative spectrum of Mg-Ca carbonate in the veinlets. The band at 1099 cm^-1^ is characteristic of dolomite (RRUFF- R050129.2). C) Representative pyroaurite spectrum in the serpentine – magnetite – dolomite vein. Bands at 145, 301, 386, 460, 530, and 1060 cm^-1^ are consistent with pyroaurite (RRUFF – R100053).


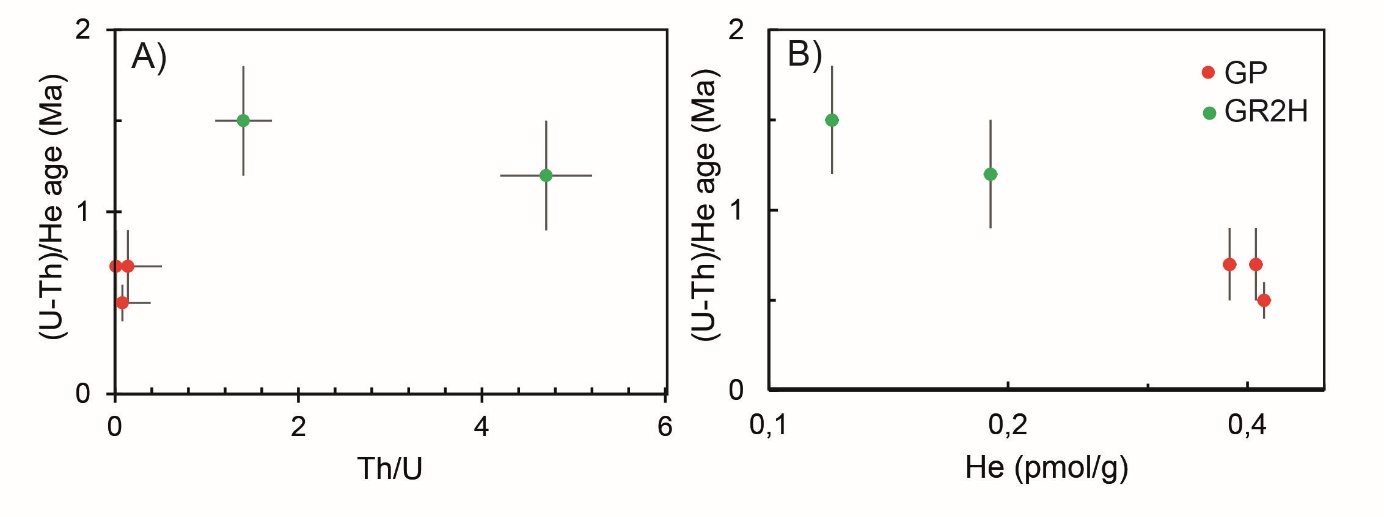
**Figure S8.** Evolution of the MgHe age as a function of **A)** the Th/U ratio and **B)** the He content in pmol/g.


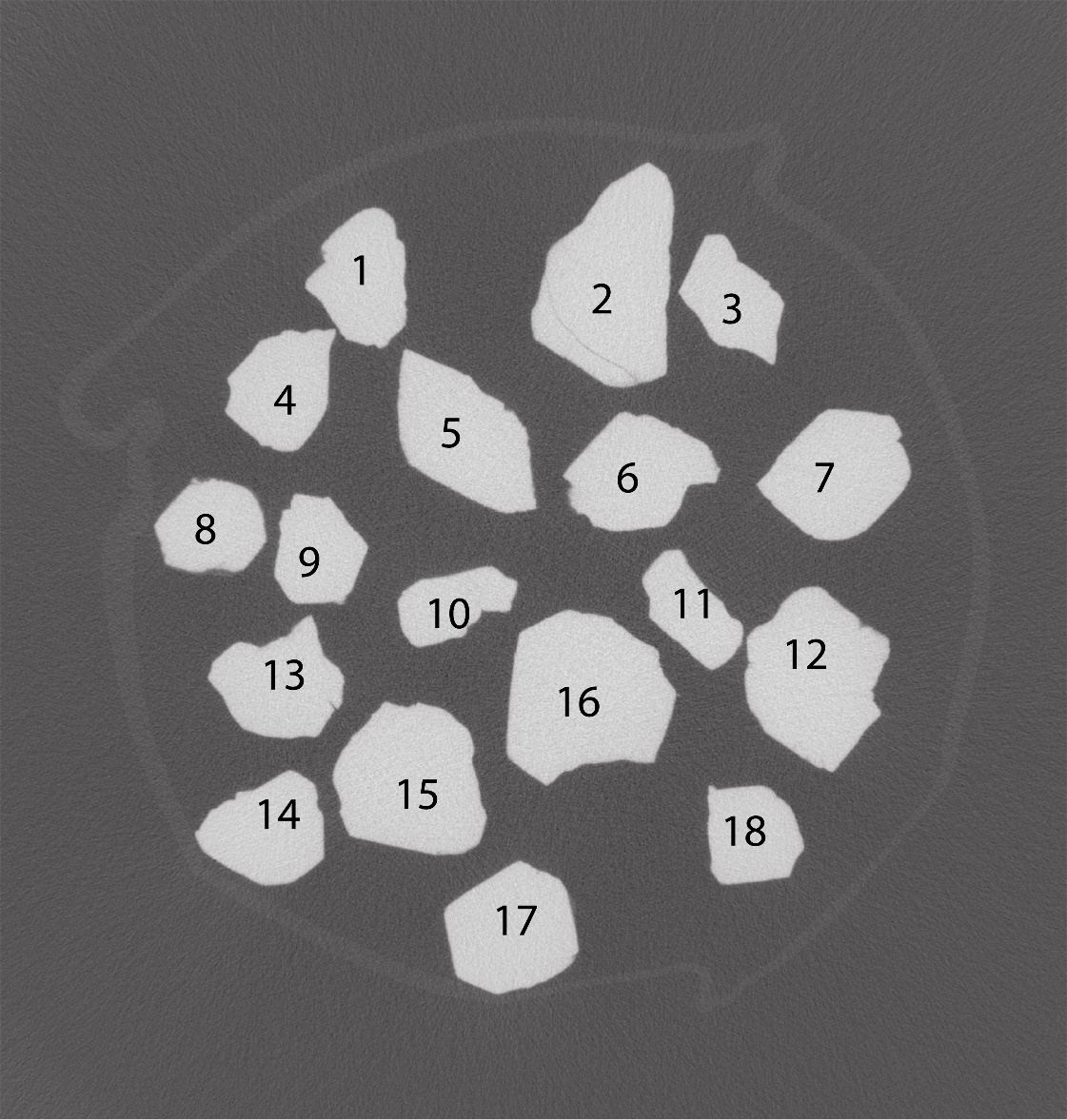


200 µm

**Figure S9.** Computed tomography scan (CT scan) image of GR2H magnetite grains. Grains #2 and #13 show the presence of cracks and were thus discarded for further MgHe dating.

**Figure S10.** BSE image of GR2H sample pieces with location of the SIMS spots. Dolomite: δ^13^C (red dots) and δ^18^O (blue dots), Magnetite: δ^18^O (black dots). Zones 1 and 2 include the
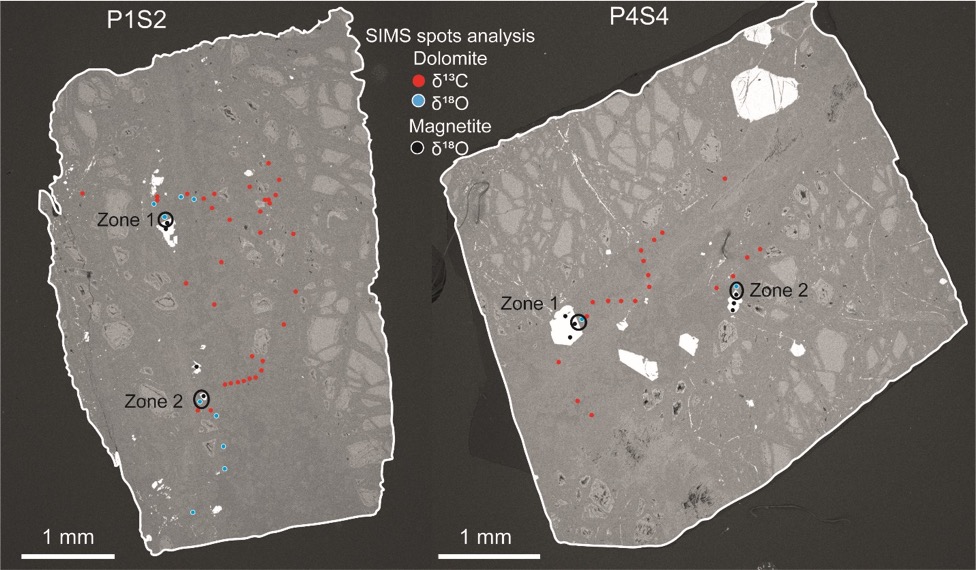
magnetite – dolomite pairs from which δ^18^O fractionation temperatures were retrieved (Table S6).


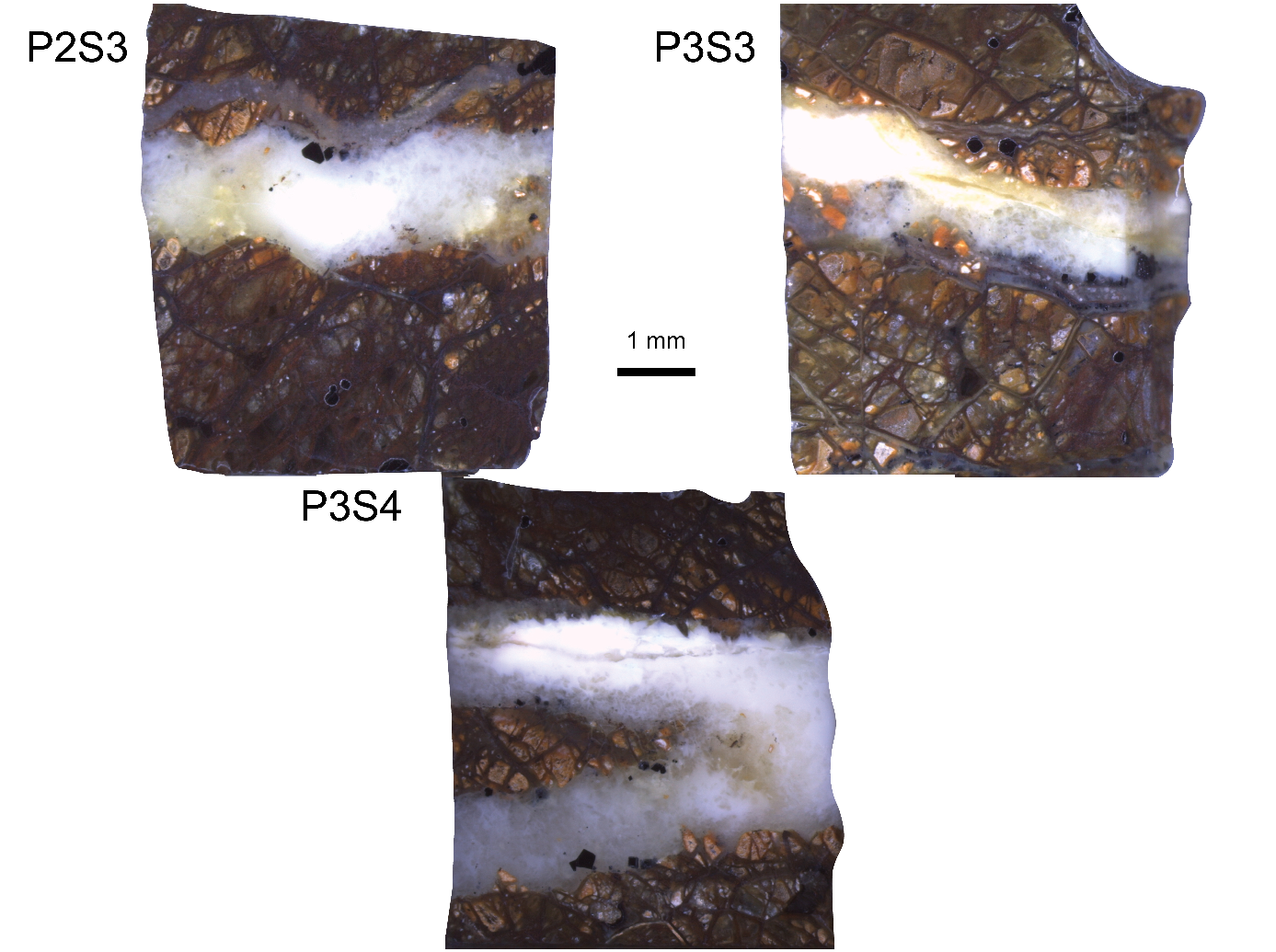


**Figure S11.** Micro-photographs of the GR2H sample areas where vein material was collected with a micro-drill in order to analyze the δ^13^C and the δ^18^O of dolomite using the micro-bulk method (Table S5).

**Supplementary Tables**

**Table S1.** Element content in wt. % (EPMA) in magnetite from Georges Pile (GP) and GR2H, and in serpentine and dolomite from GR2H.

|  | magnetite | | | | | serpentine (GR2H) | | | | dolomite (GR2H) | | spinel (GP) | |
| --- | --- | --- | --- | --- | --- | --- | --- | --- | --- | --- | --- | --- | --- |
|  | GP | | | GR2H | | host peridotite | | veins | | GR2H | | mean s.d.  (wt %) (wt%)  n=135 | |
|  | mean (wt%) | | s.d. (wt%) | mean (wt%) | s.d. (wt%) | mean (wt%) | s.d. (wt%) | mean (wt%) | s.d. (wt%) | mean (wt%) | s.d. (wt%) |  |  |
|  | n=308 |  | | n=74 |  | n=4 |  | n=9 |  | n= 128 |  |  |  |
| SiO_2_ | 0.14 | 0.03 | | 0.33 | 0.28 | 41.22 | 2.96 | 41.68 | 0.59 | - | - | 0.03 | 0.13 |
| TiO_2_ | 0.00 | 0.00 | | 0.02 | 0.01 | 0.00 | 0.00 | 0.00 | 0.00 | - | - | 0.10 | 0.01 |
| Al_2_O_3_ | 0.00 | 0.01 | | 0.00 | 0.00 | 0.38 | 0.22 | 0.13 | 0.04 | - | - | 19.55 | 2.46 |
| FeO | 88.69 | 0.81 | | 89.7 | 1.45 | 5.87 | 2.70 | 1.28 | 0.32 | 0.22 | 0.13 | 21.40 | 2.37 |
| MnO | 0.99 | 0.71 | | 0.45 | 0.23 | 0.02 | 0.04 | 0.04 | 0.02 | 2.96 | 2.12 | 0.27 | 0.03 |
| MgO | 0.76 | 0.15 | | 0.98 | 0.29 | 35.16 | 3.87 | 38.85 | 0.50 | 23.41 | 0.92 | 10.87 | 1.45 |
| Cr_2_O_3_ | 0.00 | 0.00 | | 0.00 | 0.01 | 0.01 | 0.01 | 0.01 | 0.01 | - | - | 48.43 | 2.42 |
| NiO | 0.85 | 0.33 | | 0.44 | 0.21 | 0.18 | 0.09 | 0.10 | 0.05 | 0.01 | 1.38 | 0.05 | 0.04 |
| CoO | 0.21 | 0.07 | | 0.16 | 0.05 | - | - | - | - | 0.01 | 0.01 | - | - |
| ZnO | 0.04 | 0.03 | | 0.15 | 0.08 | - | - | - | - | - | - | 0.16 | 0.03 |
| SrO | - | - | | - | - | - | - | - | - | 0.04 | 0.02 | - | - |
| CaO | - | - | | - | - | 0.12 | 0.01 | 0.19 | 0.28 | 26.68 | 1.38 | - | - |
| Mg# |  |  | |  |  | 0.85 |  | 0.98 |  |  |  |  |  |

s.d.: standard deviation, n: number of analyses

**Table S2.** (U-Th)/He results for magnetite crystals from Massif du Sud (GR2H and Georges Pile, GP). The He error is about 2% and those of U and Th is about 20%^7^. eU is the effective uranium content (eU=U+0.238×Th+0.0012×Sm^8^).

| Package name | Site | Nb of crystals | Mass (mg) | ^4^He  (10^-4^ × pmol) | ^4^He±s | ^238^U (ng) | ^238^U | ^232^Th (ng) | ^232^Th | ^147^Sm (ng) | ^147^Sm | eU (ng/g) | Th/U | Age |
| --- | --- | --- | --- | --- | --- | --- | --- | --- | --- | --- | --- | --- | --- | --- |
|  |  |  |  |  | (pmol/g) |  | (ng/g) |  | (ng/g) |  | (ng/g) |  |  | (Ma)^£^ |
| AIVa | GR2H | 4 | 1.51 | 1.81 | 0.12±0.002 | 0.016 | 10.6±2.9 | 0.023 | 15.1±3.2 | 0.001 | 0.5±0.5 | 14.3 | 1.4 | 1.5±0.3 |
| CIIa | GR2H | 5 | 1.05 | 1.68 | 0.19±0.004 | 0.012 | 11.2±1.9 | 0.056 | 52.9±5.5 | 0.001 | 0.6±0.4 | 23.9 | 4.7 | 1.2±0.3 |
| 7NC2  mag | GP | 3 | 1.38 | 5.80 | 0.42±0.008 | 0.231 | 167.7±40.2 | 0.019 | 13.9±3.3 | 0.003 | 1.8±0.4 | 171 | 0.08 | 0.5±0.1* |
| 10NC  mag | GP | 6 | 1.19 | 4.88 | 0.41±0.008 | 0.121 | 101.8±24.4 | 0.001 | 0.6±0.1 | 0.0001 | 0.1±0.02 | 102 | 0.01 | 0.7±0.2* |
| 11NC  mag | GP | 11 | 1.05 | 3.99 | 0.38±0.008 | 0.104 | 99.1±23.8 | 0.016 | 13.5±3.2 | 0.0002 | 1.0±0.2 | 102 | 0.14 | 0.7±0.2 * |

* In this study, the U and Th content are determined using quadrupole ICP-MS allowing to determine the ^238^U and ^230^Th content^9^. The (U-Th)/He age is calculated assuming U-Th-series disequilibrium of the alpha decay chains. For ages below 0.8 Ma that is the U-Th series equilibrium time, the calculated ages could be younger as it is the case for GP MgHe crystals. The age of the aliquot 7NC2mag of 0.5±0.1 Ma could be younger by a few percent.

£ The raw (U-Th)/He ages are not corrected from alpha ejection^10^ due to the large crystal sizes (400 μm), and the possible effect of U-Th zoning on the He budget was not considered^11^. However, U-Th zoning can modify the MgHe ages by no more than ± 20-40 %^12^. The term “Quaternary age” will be used since it encompasses the ages measured here with their uncertainty which includes the impact of U-Th series disequilibrium and possible impact on U-Th zoning on MgHe ages.

**Table S3.** δ^18^O composition of dolomite (Dol; n=27) and magnetite (Mag; n=26) in the GR2H veinlets obtained by *in situ* SIMS on three samples (P1S2, P1S4 and P4S4).

| P1S2 | δ^18^O SMOW (‰) | | δ^18^O SMOW (‰) | | P1S4 | δ^18^O SMOW (‰) | | δ^18^O SMOW (‰) | | P4S4 | δ^18^O SMOW (‰) | | δ^18^O SMOW (‰) | |
| --- | --- | --- | --- | --- | --- | --- | --- | --- | --- | --- | --- | --- | --- | --- |
| n°spot | Dol | 2σ (‰) | Mag | 2σ (‰) | n°spot | Dol | 2σ (‰) | Mag | 2σ (‰) | n°spot | Dol | 2σ (‰) | Mag | 2σ (‰) |
| 1 |  |  | -13.1 | 0.4 | 1 |  |  | -12.2 | 0.5 | 1 |  |  | -13.2 | 0.5 |
| 2 |  |  | -13.5 | 0.4 | 2 |  |  | -12.5 | 0.4 | 2 |  |  | -12.8 | 0.5 |
| 3 | 19.7 | 0.4 |  |  | 3 |  |  | -12.7 | 0.4 | 3 |  |  | -12.5 | 0.5 |
| 4 | 21.7 | 0.4 |  |  | 4 |  |  | -12.2 | 0.5 | 4 | 20.7 | 0.8 |  |  |
| 5 | 21.8 | 0.4 |  |  | 5 |  |  | -12.8 | 0.5 | 5 |  |  | -10.6 | 0.6 |
| 6 | 20.5 | 0.4 |  |  | 6 |  |  | -12.4 | 0.4 | 6 |  |  |  | 0.5 |
| 7 | 21.2 | 0.4 |  |  | 7 |  |  | -12.4 | 0.4 | 7 |  |  |  | 0.5 |
| 8 |  |  | -11.9 | 0.4 | 8 |  |  | -12.6 | 0.4 | 8 |  |  |  | 0.5 |
| 9 |  |  | -12.2 | 0.4 | 9 | 20.9 | 0.4 |  |  | 9 |  |  |  | 0.5 |
| 10 | 20.6 | 0.4 |  |  | 10 | 21.4 | 0.4 |  |  | 14 |  |  | -14.2 | 0.5 |
| 11 | 21.2 | 0.4 |  |  | 11 | 21.2 | 0.4 |  |  | 15 |  |  | -12.8 | 0.5 |
| 12 | 20.7 | 0.4 |  |  | 12 | 20.9 | 0.4 |  |  | 16 |  |  | -12.3 | 0.5 |
| 13 | 20.9 | 0.4 |  |  | 13 |  |  | -10.4 | 0.4 | 17 | 21.2 | 0.8 |  |  |
| 14 | 19.6 | 0.4 |  |  | 14 | 21.0 | 0.4 |  |  |  |  |  |  |  |
|  |  |  |  |  | 15 | 20.4 | 0.4 |  |  |  |  |  |  |  |
|  |  |  |  |  | 16 | 20.9 | 0.4 |  |  |  |  |  |  |  |
|  |  |  |  |  | 17 |  |  | -12.3 | 0.4 |  |  |  |  |  |
|  |  |  |  |  | 18 | 21.0 | 0.4 |  |  |  |  |  |  |  |
|  |  |  |  |  | 19 |  |  |  | 0.4 |  |  |  |  |  |
|  |  |  |  |  | 20 | 21.7 | 0.4 |  |  |  |  |  |  |  |
|  |  |  |  |  | 21 |  |  | -11.7 | 0.4 |  |  |  |  |  |
|  |  |  |  |  | 22 | 21.7 | 0.4 |  |  |  |  |  |  |  |
|  |  |  |  |  | 23 |  |  | -11.3 | 0.4 |  |  |  |  |  |
|  |  |  |  |  | 24 | 21.7 | 0.4 |  |  |  |  |  |  |  |
|  |  |  |  |  | 25 | 20.7 | 0.4 |  |  |  |  |  |  |  |
|  |  |  |  |  | 26 | 21.6 | 0.4 |  |  |  |  |  |  |  |
|  |  |  |  |  | 27 |  |  | -10.8 | 0.4 |  |  |  |  |  |
|  |  |  |  |  | 28 |  |  | -11.7 | 0.4 |  |  |  |  |  |
|  |  |  |  |  | 29 | 20.9 | 0.4 |  |  |  |  |  |  |  |
|  |  |  |  |  | 30 |  |  | -11.8 | 0.4 |  |  |  |  |  |
|  |  |  |  |  | 31 | 21.1 | 0.4 |  |  |  |  |  |  |  |

**Table S4.** δ^13^C composition of dolomite (n= 74) in GR2H veins obtained by SIMS on three samples (P1S2, P1S4 and P4S4).

| P1S4 | δ^13^C PDB (‰) | | P1S2 | δ^13^C PDB (‰) | | P4S4 | δ^13^C PDB (‰) | |
| --- | --- | --- | --- | --- | --- | --- | --- | --- |
| n°spot | Dol | 2σ  (‰) | n°spot | Dol | 2σ (‰) | n°spot | Dol | 2σ (‰) |
| 1 | 10.8 | 0.6 | 1 | 15.4 | 0.5 | 1 | 13.0 | 0.5 |
| 2 | 11.9 | 0.5 | 2 | 13.1 | 0.5 | 2 | 12.9 | 0.5 |
| 3 | 13.8 | 0.5 | 3 | 14.9 | 0.4 | 3 | 12.2 | 0.6 |
| 4 | 11.6 | 0.5 | 4 | 15.3 | 0.5 | 4 | 10.1 | 0.5 |
| 5 | 15.8 | 0.5 | 5 | 13.8 | 0.5 | 5 | 13.4 | 0.4 |
| 6 | 16.2 | 0.5 | 6 | 13.5 | 0.5 | 6 | 13.4 | 0.5 |
| 7 | 14.9 | 0.5 | 7 | 15.2 | 0.6 | 7 | 12.1 | 0.5 |
| 8 | 15.1 | 0.5 | 8 | 13.9 | 0.5 | 8 | 12.1 | 0.5 |
| 11 | 12.4 | 0.5 | 9 | 12.0 | 0.5 | 9 | 13.3 | 0.6 |
| 12 | 14.7 | 0.5 | 10 | 14.9 | 0.5 | 10 | 12.7 | 0.5 |
| 13 | 14.8 | 0.5 | 11 | 14.8 | 0.5 | 11 | 12.6 | 0.5 |
| 14 | 14.6 | 0.5 | 12 | 15.2 | 0.5 | 12 | 10.8 | 0.5 |
| 15 | 13.7 | 0.4 | 13 | 15.5 | 0.5 | 13 | 12.4 | 0.5 |
| 16 | 14.7 | 0.5 | 14 | 12.5 | 0.4 | 14 | 13.0 | 0.6 |
| 17 | 15.9 | 0.6 | 15 | 13.4 | 0.5 | 15 | 13.8 | 0.4 |
| 18 | 10.2 | 0.4 | 16 | 14.1 | 0.4 | 16 | 13.8 | 0.6 |
| 19 | 13.9 | 0.5 | 17 | 15.1 | 0.4 | 17 | 11.9 | 0.7 |
| 22 | 16.6 | 0.5 | 18 | 8.0 | 0.5 | 18 | 13.9 | 0.5 |
| 23 | 16.5 | 0.5 | 19 | 15.5 | 0.5 | 19 | 13.6 | 0.6 |
| 24 | 15.8 | 0.5 | 20 | 15.3 | 0.5 | 20 | 12.6 | 0.5 |
| 25 | 17.3 | 0.5 | 24 | 7.1 | 0.5 |  |  |  |
| 26 | 14.8 | 0.5 | 25 | 10.2 | 0.4 |  |  |  |
| 27 | 16.3 | 0.5 |  |  |  |  |  |  |
| 28 | 7.6 | 0.5 |  |  |  |  |  |  |

**Table S5.** Oxygen and carbon isotopic composition of dolomite (micro-bulk). Samples depicted in Figure S11.

| Sample Name | δ^18^O SMOW (‰) | 2σ  (‰) | δ^13^C PDB (‰) | 2σ  (‰) |
| --- | --- | --- | --- | --- |
| P2S3 | 22.7 | 0.16 | 6.3 | 0.06 |
| P3S2 | 21.4 | 0.16 | 10.7 | 0.06 |
| P3S4 | 22.6 | 0.16 | 11.5 | 0.06 |

**Table S6.** δ^18^O temperatures derived from equilibrium fractionation between magnetite – dolomite pairs as showed on Fig S10. *MAD* stands for Median Absolute Deviation.

| Sample name | Zone | SIMS spots | δ^18^O ‰ | | $1000ln\alpha_{Mag-Dol}$ | Equilibrium temperature |
| --- | --- | --- | --- | --- | --- | --- |
|  |  |  | Magnetite | Dolomite | ‰ | °C |
| P1S2 | Zone 1 |  | -13.5 | 20.5 | -33.9 | 93.5 |
|  | Zone 2 |  | -11.9 | 21.2 | -33.1 | 98.5 |
| P1S4 | Zone 1 | 1 | -12.2 | 20.9 | -33.1 | 98.5 |
|  |  | 2 | -12.5 | 21.4 | -33.9 | 93.5 |
|  |  | 3 | -12.7 | 21.2 | -33.9 | 93.5 |
|  |  | 4 | -12.2 | 20.9 | -33.1 | 98.5 |
|  | Zone 2 | 1 | -10.8 | 20.9 | -31.7 | 110.0 |
|  |  | 2 | -11.8 | 21.1 | -32.9 | 100.0 |
|  | Zone 3 | 1 | -10.4 | 20.4 | -30.8 | 117.0 |
|  | Zone 4 | 1 | -12.3 | 21.0 | -33.3 | 97.0 |
|  | Zone 5 | 1 | -11.7 | 21.7 | -33.4 | 96.5 |
|  | Zone 6 | 1 | -11.3 | 21.7 | -33.0 | 99.5 |
| P4S4 | Zone 1 | 1 | -12.8 | 20.7 | -33.6 | 95.0 |
|  | Zone 2 | 1 | -12.8 | 21.2 | -33.9 | 93.5 |
|  |  |  |  |  | **Median** | **97** |
|  |  |  |  |  | **2 x MAD** | **5** |

**Thermodynamic data for the phases added to the LLNL PHREEQC database**

Ferro-brucite_MC

Fe(OH)2 + 2.0000 H+ = 1.0000 Fe++ + 2.0000 H2O

log_k 12.715

-analytic 6.7795147 -0.0951366 1.0380489 11.411864 0.5001865 0.0000657

-Vm 30.5 cm3/mol

Pyroaurite_Rozov

Mg3Fe(OH)8(CO3)0.5(H2O)2.5 = 3.000 Mg+2 + 1.00 Fe+3 + 8 OH- + 0.5 CO3-2 + 2.5 H2O

log_k -68.04

-analytic 543.36 0.0 -30562.50 -205.6669 0.0 0.0

-Vm 119.0 cm3/mol

**References cited in the Supplemental Material**

1. O’Neil, J. R. Theoretical and experimental aspects of isotopic fractionation. *Rev. Mineral*. **16**, 1-40. https://doi.org/10.1515/9781501508936-006 (1986).
2. Chacko, T., Cole, D. R. & Horita, J. Equilibrium oxygen, hydrogen and carbon isotope fractionation factors applicable to geologic systems. *Rev. Mineral. Geochem*. **43**, 1-81. https://doi.org/10.1515/9781501508745-004 (2011).
3. Zheng, Y.-F. On the theoretical calculations of oxygen isotope fractionation factors for carbonate-water systems. *Geochem. J*. **45**, 341-354. https://doi.org/10.2343/geochemj.1.0125 (2011).
4. Warr, L. N. IMA–CNMNC approved mineral symbols. *Min. Mag*. **85**(3), 291-320. <https://doi.org/10.1180/mgm.2021.43> (2021).
5. Cluzel, D. *et al.* Late Oligocene post-obduction granitoids of New Caledonia: A case for reactivated subduction and slab break-off. *Isl. Arc* **14**, 254–271. https://doi.org/10.1111/j.1440-1738.2005.00470.x (2005).
6. Schwartz, S. *et al.* Pressure–temperature estimates of the lizardite/antigorite transition in high pressure serpentinites. *Lithos* **178**, 197-210. http://dx.doi.org/10.1016/j.lithos.2012.11.023 (2013).
7. Corre, M. *et al.* U and Th content in magnetite and Al spinel obtained by wet chemistry and laser ablation methods: implication for (U–Th)∕ He thermochronometer. *Geochronol*. **4**, 665-681. https://doi.org/10.5194/egusphere-2022-520 (2022).
8. Cooperdock, E. H. G., Ketcham, R. A. & Stockli, D. F. Resolving the effects of 2-D versus 3-D grain measurements on apatite (U-Th)/He age data and reproducibility. *Geochronol.* **1**, 17-41. https://doi.org/10.5194/gchron-1-17-2019 (2019).
9. Gautheron, C. *et al*. Technical note: Analytical protocols and performance for apatite and zircon (U–Th)/He analysis on quadrupole and magnetic sector mass spectrometer systems between 2007 and 2020. *Geochronol.* **3**, 351-370 (2021).
10. Ketcham, R.A., Gautheron, C. & Tassan-Got, L. Accounting for long alpha-particle stopping distances in (U-Th-Sm)/He geochronology: refinement of the baseline case. *Geochim. Cosmochim. Acta* **75**, 7779-7791 (2011).
11. Hourigan, J.K., Reiners, P.W. & Brandon, M.T. U-Th zonation-dependent alpha-ejection in (U-Th)/He chronometry. *Geochim. Cosmochim. Acta* **69**, 3349-3365 (2005)
12. Gautheron, C. *et al.* Accounting for long alpha-particle stopping distances in (U-Th-Sm)/He geochronology: 3D modeling of diffusion, zoning, implantation, and abrasion. *Geochim. Cosmochim. Acta* **96**, 44-56 (2012).
